# Fungicidal action of geraniol against *Candida albicans* is potentiated by abrogated CaCdr1p drug efflux and fluconazole synergism

**DOI:** 10.1371/journal.pone.0203079

**Published:** 2018-08-29

**Authors:** Shweta Singh, Zeeshan Fatima, Kamal Ahmad, Saif Hameed

**Affiliations:** 1 Amity Institute of Biotechnology, Amity University Haryana, Gurugram (Manesar), India; 2 Center for Interdisciplinary Research in Basic Sciences, Jamia Millia Islamia, New Delhi, India; Louisiana State University, UNITED STATES

## Abstract

Among the several mechanisms of multidrug resistance (MDR), overexpression of drug efflux pumps CaCdr1p and CaMdr1p belonging to ATP binding cassette (ABC) and major facilitator superfamily (MFS) respectively remain the predominant mechanisms of candidal infections. Therefore inhibiting or modulating the function of these transporters continues to draw attention as effective strategy to combat MDR. We have previously reported the antifungal potential of Geraniol (Ger), a natural monoterpenoid from Palmarosa oil, against *Candida albicans*. Herein, we explored the fungicidal nature of Ger. The Rhodamine 6G (R6G) and Nile red accumulation confirms the specific effect on CaCdr1p. Mechanistic insights with *Candida* cells overexpressing CaCdr1p and CaMdr1p revealed that Ger specifically modulates CaCdr1p activity. Kinetic studies further unraveled the competitive inhibition of Ger for R6G efflux as evident from increased apparent Km without affecting V_max_ value. The effect of Ger on CaCdr1p was substantiated by molecular docking analyses, which depicted in-silico binding affinity of Ger with CaCdr1p and explored that Ger binds to the active site of CaCdr1p with higher binding energy. Although RT-PCR and western blot revealed no change in expressions of *CDR1* and CaCdr1p, confocal microscopy images however depicted CaCdr1p mislocalization in presence of Ger. Interestingly, Ger was synergistic (FICI<0.5) with fluconazole (FLC) which is a well known antifungal drug. Furthermore, Ger sensitizes the FLC sensitive and resistant clinical matched pair of isolates Gu4/Gu5 and led to abrogated R6G efflux and depleted ergosterol. Furthermore, Rhodamine B labeling demonstrates altered mitochondrial potential with Ger which suggest possible linkage of dysfunctional mitochondria with CaCdr1p activity. We also estimated phenotypic virulence marker extracellular phospholipase activity which was considerably diminished along with inhibited cell adherence and biofilm biomass. Lastly, antifungal efficacy of Ger was demonstrated by enhanced survival of *Caenorhabditis elegans* model and negligible hemolytic activity (20%). Together, modulation of efflux pump activity by Ger and FLC synergism represent a promising approach for combinatorial treatment of candidiasis.

## Introduction

In the recent times, fungal infections have increased at an alarming rate in the immunocompromised patients with transplantation, cancer chemotherapies, surgery, HIV infections etc [1]. Among many fungal genera, *Candida* species are the most prominent causing superficial to invasive mucosal infections leading to disseminated systemic infections [2,3]. Azoles (fluconazole) are most commonly prescribed antifungal drugs for candidal infections as they have low toxicity and more bioavailability. However, the prolonged use of fluconazole (FLC) along with other antifungal drugs (polyenes and echinocandins) results in drug resistance by a phenomenon of Multi drug resistance (MDR) [4, 5, 6]. MDR is a multifactorial phenomenon due to amalgamation of various factors leading to drug resistance. Many biochemical studies have highlighted significant diversity of the mechanisms conferring resistance to azoles [7]. Among the resistance mechanisms, overexpression of efflux pumps belonging to class ATP binding Cassette (ABC) and major facilitator superfamily (MFS) has been mainly implicated in azole resistance [8, 9]. The genes encoding these integral membrane proteins are *Candida* drug resistance, *CDR1* and *CDR2* and multidrug resistance (*MDR1*). As they are major players of drug resistance mechanism, they are effective targets for combating MDR either by blocking them structurally or functionally [10]. Thus, there is an imminent need to search for new antifungal agents that can block or modulate the functionality of drug efflux pumps for treating *C*. *albicans* infection.

Coincidentally, the identification or development of new antifungal drugs needs long time and huge economic investment. Therefore, much attention has been paid in search for phytotherapeutics of natural origin. Infact, to solve this problem, many natural inhibitors have been identified which can affect or modulate the activity of MDR transporters [11]. The natural compounds can either inhibit the efflux functions or act synergistically with the known antifungal drugs. Curcumin is a natural product from turmeric, which has been reported to modulate the CaCdr1p activity synergies with FLC [12]. Plagiochin E, a bisbibenzyl phenolic in nature has shown the downregulation of the *CDR1* gene which can also work synergistically with FLC [13]. Jatrophane diterpenoid euphopubescenol which is extracted from *Euphorbia pubescens* has been shown to inhibit the CaMdr1 efflux pump protein by binding to the protein [14]. Geraniol (Ger) belongs to the class of terpenes (acyclic monoterpenoid) which is formed by condensation of isoprene units, is a main component of geranium oil, palmorsa oil and known for antibacterial, antihelmintic, anti-inflammatory and anticarcinogenic properties [15]. Previous study has revealed antifungal potential and diverse cellular targets of Ger against *C*. *albicans* [16]. Notably, the effect of Ger on fungal drug transporters is not known. In this study, to gain further mechanistic insights, we have elucidated the inhibitory effect of Ger on CaCdr1p. Additionally, this study for the first time establishes that Ger is fungicidal in nature and confers synergism with FLC. We also demonstrated that Ger reverses the FLC resistance of clinical resistant isolates. Further effect of Ger on extracellular phopholipase activity, cell adherence and biofilm biomass has also been demonstrated. The *in-vivo* efficacy of Ger is depicted by using *Caenorhabditis elegans* nematode infection model.

## Materials and methods

### Materials

All Media chemicals including YPD (Yeast Extract Peptone Dextrose), nutrient broth, rhodamine 6G (R6G), 2-deoxy glucose (2-DOG), 2,4 dinitrophenol (2,4 DNP), n- heptane, peptone, brain heart infusion (BHI) media, rhodamine B, Nile red (NR), Tris base, agarose, bovine serum albumin (BSA), cholesterol, ampicillin and Egg yolk were purchased from Himedia Laboratories (Mumbai, India). Sodium chloride (NaCl), calcium chloride (CaCl_2_), ammonium chloride (NH_4_Cl), magnesium chloride (MgCl_2_) potassium chloride (KCl), mannitol, di-sodium hydrogen orthophosphate (Na_2_HPO_4_), potassium di-hydrogen orthophosphate (KH_2_PO_4_), di-potassium hydrogen orthophosphate (K_2_HPO_4_), sodium hydroxide (NaOH), D-glucose, sodium dodecyl sulphate (SDS), potassium hydroxide (KOH), ammonium persulphate (NH_4_)_2_S_2_O_8_) dimethyl sulphoxide (DMSO), acrylamide, bis-acrylamide, glycerol, bromophenol blue, TEMED, glycine, copper sulphate (CuSo_4_), sodium carbonate (Na_2_CO_3_), sodium potassium tatarate (KNaC_4_H_4_O_6_·4H_2_O), Tween 20 were obtained from Thermo Fischer Scientific, India. Calcofluor white (CFW), Geraniol (Ger), flucanozole (FLC), Amphotericin B (Amp B), caspofungin (CAS), diethyl pyrocarbonate (DEPC), 4-morpholinepropanesulfonic acid (MOPS), tri-reagent, DNase were obtained from Sigma-Aldrich Chemical Co. (St. Louis, MO, USA). Nitrocellulose membrane and ECL kit was purchased from Bio-Rad laboratories Pvt. Ltd., India.

### Yeast strains and growth media

The strains used in this study are listed in (S1 Table). All the strains of *C*. *albicans* were cultured in YPD broth with the composition of yeast extract 1% (w/v), peptone 2% (w/v) and dextrose 2% (w/v). For agar plates 2% (w/v) agar was added to the medium. To check the persistence of *Candida* cells in *C*. *elegans*, Act1p-GFP was grown in brain heart infusion (BHI) media and fed to *C*. *elegans*. All *Candida* strains were stored in 30% (v/v) glycerol stock at −80°C. The cells were freshly revived on YPD broth and transferred to agar plate before each study to ensure the revival of the strains. For biochemical assays, Ger (dissolved in DMSO) was used at its subinhibitory concentration as described previously [16]

### Static /Cidal determination assay

For the static-cidal assay three replicate cultures were prepared and *Candida* cells were seeded into each culture tube at 0.1 OD_600_ in the absence and presence of Ger and all cultures were incubated at 30°C. Next day, 100μl cultures are drawn and re-inoculated into the fresh YPD media without Ger and incubated for 24hrs and OD_600_ was measured with spectrophotometer. For culture plates, fresh cells were streaked by quadrant streak method on the YPD media plate and other one supplemented with Ger at its MIC value and incubated at 30°C for 24 hrs. Next day, the revived cells were re-streaked on the fresh YPD plates without Ger and reincubated for 24 hrs. Next day, the plates were checked for any growth.

### R6G and NR intracellular accumulation

Accumulation assays were performed according to previously described protocol with minor modifications [17, 18]. Briefly, cells were grown overnight in YPD medium at 30°C with constant agitation and further incubated for 2 hr at 30°C in the presence of 135μg/ml of Ger. After incubation, cells were harvested by centrifugation at 5000 x g at 4° C for 5 min, washed twice with 10 mM phosphate buffer solution (PBS) at pH 7.2. Then, 2x10^6^ cells/ml were resuspended in 1.0 ml PBS containing 2% glucose plus R6G (final concentration of 4 μM) or NR (final concentration of 7 μM) and incubated for 30 min at 30°C. After that, cells were harvested by centrifugation at 5000x g at 4° C for 5 min and washed twice with cold PBS. The final pellet was collected for fluorescence microscopy analysis.

### R6G extracellular efflux assay

The efflux of R6G was determined essentially using a previously described protocol [19]. Briefly, approximately 1x10^6^ yeast cells from an overnight-grown culture were transferred to YPD medium and allowed to grow for 5 h in presence of Ger. Cells were pelleted, washed twice with phosphate-buffered saline (PBS) (without glucose), and resuspended as a 2% cell suspension, corresponding to 10^8^ cells (wt/vol) in PBS without glucose. The cells were then de-energized for 45 min in 2-DOG (5 mM) and 2,4 DNP (5 mM) in PBS (without glucose). The de-energized cells were pelleted, washed, and resuspended as a 2% cell suspension (wt/vol) in PBS without glucose, to which R6G was added at a final concentration of 10 μM and incubated for 40 min at 30°C. The equilibrated cells with R6G were then washed and resuspended as a 2% cell suspension (wt/vol) in PBS without glucose. Samples with a volume of 1 ml were withdrawn at the indicated time and centrifuged at 9,000 g for 2 min. The supernatant was collected, and OD was measured at 527 nm. Energy dependent efflux (at the indicated time) was measured after the addition of glucose (2%) to the cells resuspended in PBS (without glucose). Glucose-free controls were included in all the experiments. For competition assays, Ger (135μg/ml) was added to the de-energized cells 5 min before the addition of R6G and allowed to equilibrate.

### Docking studies

#### 3D structure prediction by I-TASSER and validation

The sequence of 1500 amino acids of *C*. *albicans* drug resistance protein 1 (CaCdr1p) was retrieved from Swiss Prot database in FASTA format "S1 Fig". The three dimensional model was generated using Modeller and I-TASSER server which generates 3D model of query sequence by multiple threading alignments and iterative structural assembly simulation [20, 21]. I-TASSER methodology includes general steps of threading, structural assembly, model selection, refinement, and structure based functional annotation as described previously reported [22].

#### Validation of the predicted model

The conformation of best model of CaCdr1p predicted by I-TASSER was validated by Ramachandran plot. The conformation of the predicted model was calculated by analyzing the phi (Φ) and psi (Ψ) torsion angles using PROCHECK online server. The assessment of model was also confirmed by rechecking through MolProbity online server.

#### Prediction of active site of the model

The active sites of CaCdr1p target proteins were identified by using our earlier reported method through computed atlas of surface topography of proteins (CASTp) server (http://cast.engr.uic.edu) [22].

#### Preparation of ligands and protein molecule

The hydrogen atoms having polar nature were added to the I-TASSER predicted model of CaCdr1p, the residue structures having inferior tenancy were deleted, and the side chains that were incomplete were replaced by using Auto Dock Tools (ADT) version 1.5.6 from the Scripps Research Institute. Further, to each atom having Gasteiger charges were added and the non-polar hydrogen atoms were merged to the protein structure. Then the structures constructed were saved in PDBQT file format, for further analysis in ADT [22, 23]. The next crucial step was the preparation of CaCdr1p known inhibitor and Geraniol 3D structures that were achieved using the software ChemBioDraw Office 12.0 (licensed @ Cambridge’s soft). Known inhibitors of CaCdr1p include Farnesol [24]. The PDB file of ligand was then generated and converted into PDBQT file by process like detect root, choose the torsion and set the number of torsion by using ADT [22, 23].

#### Receptor grid formation

Grid mapping is a requirement to direct protein inhibitor and ligands to look for their region of strong affinity to the protein active site. The grid dimensions for CaCdr1p protein was 54x54x52 grid points respectively with interspacing of 0.463 Å between the grid points but centered on the ligand for CaCdr1p protein (157.636, 106.336 and 162.516) coordinates. The grid was created for the search of promising interaction and best support during the docking which represents orientation, position and conformation of the receptors [25].

#### Molecular docking

Molecular docking stimulation using the ligand molecules of CaCdr1p was conducted using Autodock 4.2 docking suite by employing Lamarckian genetic algorithm as described previously [22, 23, 26]

### RT-PCR

RNA was isolated as described previously [27]. The cells were diluted into 50 ml fresh YEPD broth at OD_600_ of 0.1 (10^6^ cells ml^-1^) in absence (control) and presence of Ger (135μg/mL) and grown at 30°C till OD_600_ of 1.0. RNA isolation was performed by Trizol method and Reverse transcriptase (RT) PCR was performed as described in the RevertAid H Minus kit (Invitrogen, USA). RNA purity and integrity was assessed by A_260_/A_280_ ratio "S2 Fig" using UV-Visible spectrophotometer (VSI electronics, India). The synthesized cDNA product (2μl) was directly used for PCR amplification (50 μl) using gene specific forward and reverse primers "S2 Table". The amplified products were gel electrophoresed and the band densities (for genes of interest) were measured and quantified by normalizing to that of the constitutively expressed actin gene (*ACT1*).

### Western blotting

For protein extraction and western blotting, crude protein was extracted as described previously with slight modifications [28]. Crude protein extract was prepared from cell suspensions after 4 h of induction with Ger. Aliquots of cell suspensions were pelleted by centrifugation at 2260 × g for 5 min and resuspended in 1 mL of deionized water. Cells were lysed by adding 150 μL of 1.85 M NaOH–7.5% β-mercaptoethanol and incubated on ice for 10 min. Proteins were precipitated by adding 150 μL of 50% trichloroacetic acid and incubated on ice for 10 min. Samples were centrifuged at 10,000 × g for 5 min at 4°C, washed in 1 mL of 1 M Tris–HCl pH 8.0, and resuspended in 50 μL of sample buffer (40mM Tris–HCl, 8M urea, 5% SDS, 0.1 mM EDTA 1% β-mercaptoethanol, 0.1 mg/mL bromophenol blue). After 30 min incubation at 37°C, samples were loaded on 6% sodium dodecyl sulphate–polyacrylamide gel and developed in a Mini-PROTEAN II electrophoresis cell (Bio-Rad, India). Samples were then transferred onto nitrocellulose membrane using a Mini-PROTEAN Tetra System electrophoresis cell (Bio-Rad Laboratories Pvt. Ltd, India). Membranes were stained to check for equal loading of gels. Immunodetection of CaCdr1p was performed using polyclonal rabbit anti-GFP antibody and horseradish-peroxidase conjugated anti mouse antibody as a secondary antibody. Signals were detected using an ECL kit (Bio-Rad Laboratories Pvt. Ltd, India) as per the manufacturer’s instructions.

### Fluorescence and confocal microscopy

The overnight grown cells in presence of Ger were harvested and imaged using HL-23 Coslab Fluorescence microscope, India. The cells were stained with R6G and NR for intracellular accumulation assays, Rhodamine B for monitoring the mitochondrial membrane potential and CFW staining for biofilm biomass visualization and intestinal persistence of *C*. *elegans* fed with *C*. *albicans* [29, 30]. For confocal microscopy, the GFP tagged strains were used after 8 hr cultured cells in presence of Ger were harvested and imaged using confocal microscope, Nikon, India with 100X oil immersion objective lens to visualize the GFP-tagged strains.

### Drug susceptibility assay

#### Spot assay

Spot assays for the strains were determined using a known method as described elsewhere [16, 27]. Briefly, 5μl of fivefold serially diluted yeast cultures (cells suspended in normal saline to an OD_600_ nm of 0.1) were spotted onto YPD plates in the absence (control) and presence of the drugs. Growth was not affected by the presence of solvent used in the examination. Growth difference was measured after incubation for 48 hours at 30°C. The concentrations used in this study are specified in figure legends.

#### Checkerboard assay

The stock solutions and serial two fold dilutions of each drug with at least double the MIC were prepared according to the recommendations of NCCLS immediately prior to testing [31]. A total of 50 μl of YPD broth was distributed into each well of the microdilution plates. The first antibiotic of the combination was serially diluted along the ordinate, while the second drug was diluted along the abscissa. Each microtiter well was inoculated with 100 μl inoculum of 1 × 10^5^ CFUml^-1^, and the plates were incubated at 30°C for 48 h under aerobic conditions. The resulting checkerboard contains each combination of two antibiotics, with wells that contain the highest concentration of each antibiotic at opposite corners. According to the NCCLS guidelines for broth microdilution, the MIC was taken as the lowest concentration of antibiotic that completely inhibited growth of the organism as detected with the naked eye. Synergy is more likely to be expressed when the ratio of the concentration of each antibiotic to the MIC of that antibiotic was same for all components of the mixture. The fractional inhibitory concentration index (FICIs) were calculated as follows: FICI = FIC A + FIC B, where FIC A is the MIC of drug A in the combination/MIC of drug A alone, and FIC B is the MIC of drug B in the combination/MIC of drug B alone. The combination is considered synergistic only when the FICI is ≤0.5.

Drug potentiation was determined to check efficacy of known therapeutics in the presence of Ger. Growth media used was YPD supplemented with sub-inhibitory concentration of Ger. To check the drug efficacy 100μl of media was placed at each well of the 96 wells plate following addition of the drug with the remaining media and then serially diluted. 100μl of cell suspension (in normal saline at OD_600_ 0.1) was added to each well and OD_600_ was measured after 48 hours at 30°C. The MIC_80_ was recorded as the concentration which inhibited the growth by 80%.

#### Filter disc assay

The filter disc assay was performed as described elsewhere [27, 32]. The 5–10μL of drugs were spotted at the indicated amount in the figure legends and the diameters of the respective zones of inhibition were measured after incubation of the plates for 48 hours at 30°C.

### Quantitation of ergosterol

Sterols were extracted by the alcoholic KOH method and the percentage of ergosterol was calculated as described previously [27, 32]. Briefly, a single *C*. *albicans* colony from an overnight YPD agar plate culture was used to inoculate 50 ml of YEPD in presence and absence of Ger (135μg/ml). Both ergosterol and 24(28)-DHE absorb at 281.5 nm, whereas only 24(28)-DHE absorbs at 230 nm. Ergosterol content is determined by subtracting the amount of 24(28)-DHE (calculated from the OD_230_) from the total ergosterol plus 24(28)-DHE content (calculated from the OD_281.5_). Ergosterol content was calculated as a percentage of the wet weight of the cells with the following equations: % Ergosterol + % 24(28)-DHE = [(A281.5/290) × F] / pellet weight; % 24(28)-DHE = [(A230/518) × F] / pellet weight and % Ergosterol = [% ergosterol + % 24(28) DHE]—% 24(28) DHE, where F is the factor for dilution in petroleum ether and 290 and 518 are the E values (in percent per centimeter) determined for crystalline ergosterol and 24(28)-DHE, respectively.

### Phospholipase activity

The phospholipase activity was estimated by measuring the size of the precipitation zone (Pz) after *C*. *albicans* (90028) growth on egg yolk agar (1% peptone, 3% glucose, 5% NaCl, 0.0006% CaCl_2_, 2% agar and 10% egg yolk) as described previously [33, 34]. *C*. *albicans* (90028) were incubated in YPD liquid medium at 35°C for 24 h with treatment using drugs alone or in combination at the following concentrations: FLC at 1 μg/mL, Ger at 135μg/mL. A drug-free sample served as the control. Then, 10-μl aliquots of a suspension (10^7^ CFU/ml) were inoculated onto the egg yolk agar medium plates. The plates were incubated at 37°C for 72h, after which the Pz value was measured. Pz value was expressed as the ratio of the diameter of the colony to the diameter of the precipitation zone. The phospholipase activity was classified (Pz = 1), as very low (Pz = 0.90 to 0.99), low (Pz = 0.80 to 0.89), high (Pz = 0.70 to 0.79) and very high (Pz = 0.69), as previously reported [35]. Each experiment was performed in triplicate.

### Adherence to epithelial cells

Cell separation and adherence assays were made using a modified method [32, 36]. Briefly, yeast cells were grown on YPD overnight at 37°C and re-suspended in 2 mL of sterile PBS (pH 6.8), washed twice by centrifugation re-suspended in spider media (pH 7.2). Epithelial cells were voluntarily donated by the author via soft scraping of the cheek mucous membrane with sterile cotton swabs and gently stirred and washed with PBS by centrifugation (3000rpm, 3 min each). Adherence assays were developed by mixing 1 mL of each suspension in a test tube, followed by incubation in the presence of Ger (135μg/mL) at 37°C under gentle stirring for 2 h. After incubation, two drops of trypan blue solution (0.4%) were added to each tube and the mixture was gently shaken. 10 μL of the stained suspension were transferred to a Neubauer chamber and examined microscopically.

### Biofilm biomass quantification

Biofilm biomass was measured as described previously [37]. Preweighed sterile silicone squares (1.5×1.5 cm), were pretreated with bovine serum (Sigma) overnight and washed with PBS before inoculation. Exponentially growing *C*. *albicans* cells were diluted to an OD_600_ of 0.2 with Spider medium, and the suspension was added to a sterile 12-well plate with one prepared silicone square in each well. The inoculated plate was incubated at 37°C for 90 min with gentle agitation (150 rpm) until adhesion occurred. To remove non-adherent cells, the squares were washed with 2 ml PBS, and then moved to a fresh 12-well plate containing 2 ml fresh Spider medium. Ger (135μg/ml) was added to the fresh Spider medium. The plate was incubated at 37°C for an additional 60 h at 75 rpm agitation for biofilm formation. For dry mass measurements, the squares were washed in PBS and dried and weighed. The total biomass of each biofilm was calculated by subtracting the weight of the silicon square after biofilm formation from the preweighed silicon square.

### *C*. *elegans* studies

*C*. *elegans* nematodes were handled according to standard method [30]. Worms were grown on NGM plate at 20°C, unless otherwise stated, and routinely maintained on *E*. *coli* OP50. *C*. *elegans* were routinely purified by the bleaching method. Approximately, 40 worms at L4 stage or young adult hermaphrodites were transferred from a lawn of *E*. *coli* OP50 to BHI medium in presence of Ger (135μg/ml). Worms were considered dead when they did not respond to the tapping on the plate and scored daily for 7 days. Each experimental condition was tested in triplicate. Nematode survival was plotted using the Kaplane-Meier method.

For the *C*. *elegans* co-infection liquid assay, methodology with a few minor modifications was used as described elsewhere [22, 30]. Briefly, synchronized, young adult nematodes were sequentially preinfected with *C*. *albicans* (SC5314/Act1p-GFP) for 2 h on BHI agar medium containing ampicillin (100 mg/ml). The nematodes were then washed four times with 2 ml of sterile M9 buffer. They were collected by centrifugation at 900 rpm after each wash and pipetted into wells of a 12-well microtiter dish (NEST Pvt Ltd) containing 2 ml liquid medium (20% BHI and 80% M9 buffer) in presence of Ger (135 μg/ml). The infected *C*. *elegans* were incubated at 25°C for 3 days, and scored as live or dead on a daily basis and images were taken on the 3^rd^ day of infection. Images were taken from microscope (Olympus, India) at 10X equipped with Coslab camera.

For visualization of *Candida* cells within the worm intestine, *C*. *albicans*, SC5314 and Act1p-GFP, lawns were prepared and worms were placed for 6 h, thoroughly washed with M9 buffer, and then pipette onto OP50 seeded NGM plates. After 2 days worms were thoroughly washed again with M9 buffer. SC5314 fed *C*. *elegans* were stained with CFW on 2^nd^ day of infection. Fluorescence of Act1p-GFP and CFW stained *C*. *albicans* were observed in the proximal and distal intestine of *C*. *elegans* through HL-23 Coslab Fluorescence microscope, India [22].

### Hemolytic activity

The hemolytic activity of Ger (225μg/ml) was assayed as described elsewhere [38], with modifications. In brief, fresh human red blood cells (hRBCs) collected in the presence of an anti-coagulant from a healthy volunteer (author donated voluntarily) were washed three times in PBS. The Ger was added to the suspension of red blood cells (4%, v/v) in PBS to a final volume of 1mL and incubated at 37°C for 35 min. The samples were then centrifuged for 2 min at 2000rpm, and the release of hemoglobin was monitored by measuring the absorbance (A_sample_) of the supernatant at 540nm. For negative and positive controls, hRBCs in PBS (A_blank_) and in 1% (final concentration v/v) Triton X-100 were used, respectively. The percentage of hemolysis was calculated according to the following equation: Hemolysis (%) = [(A_sample_—A_blank_)/(A_Triton_—A_blank_)]*100.

### Statistical analyses

All experiments were performed in triplicates (n = 3). The results are reported as mean ± standard deviation (SD) and analyzed using Student’s t test where in P < 0.05 was considered as statistically significant.

## Results

### Ger is fungicidal in nature

**Fig 1:**
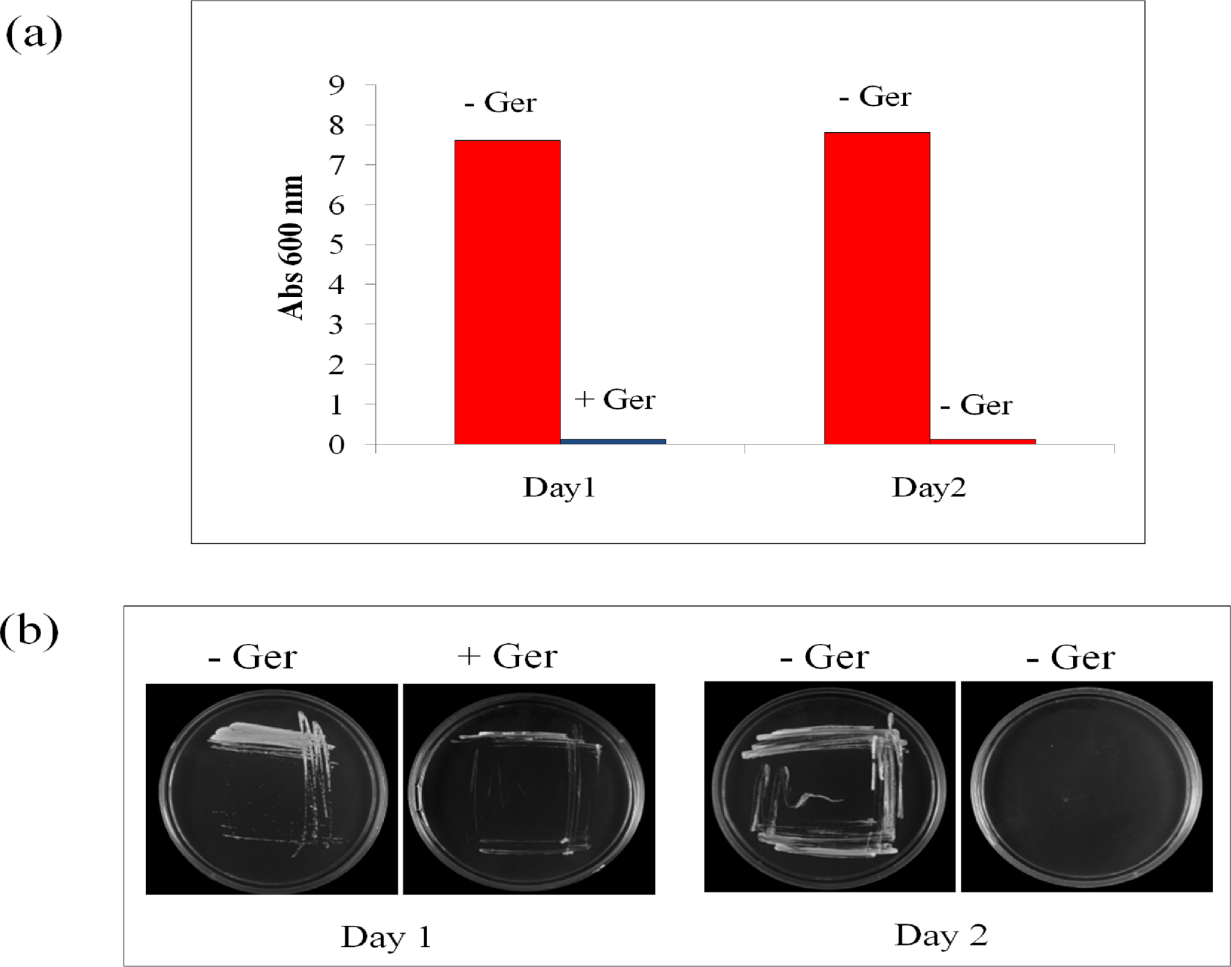
Static cidal assay of Ger. **(a)** Bar graph depicting the revival of untreated candidal cells (-Ger) and non revival of Ger treated (MIC_80_) cells in normal YPD media confirming the fungicidal nature of Ger. **(b)** Quadrant streaking on YPD plates supplemented with and without Ger depicting the revival of untreated candidal cells (-Ger) and non revival of Ger treated (MIC_80_) cells in YPD agar media reconfirming the fungicidal nature of Ger.

Previous study has shown that Ger has potent antifungal activity not only against *C*. *albicans* but also against non-*albicans* species [16]. In an attempt to probe the cidal or static nature of Ger, we performed two autonomous assays. Firstly, we did a spectrophotometric based assay where we grew the cells in the absence and presence of Ger at MIC. As expected, the growth was inhibited in Ger treated cells while the control cells grew without any restriction on day 1 "Fig 1A". When same sets of cells were re-innoculated into a fresh media without Ger, then intriguingly, there was no revival of candidal cells from the Ger treated cultures on day 2 depicting its fungicidal nature "Fig 1A". To confirm this hypothesis, through quadrant streaking method, candidal cells were streaked on the Ger (MIC) treated YPD plates along with the control YPD plates and growth was monitored after day1 of treatment "Fig 1B". As expected, the growth was unrestricted in control and inhibited in Ger containing plate, and when same cells were re-streaked in fresh YPD plates then the growth was still not recovered despite absence of Ger on day 2 "Fig 1B".

### Ger inhibits R6G and Nile red extrusion

**Fig 2:**
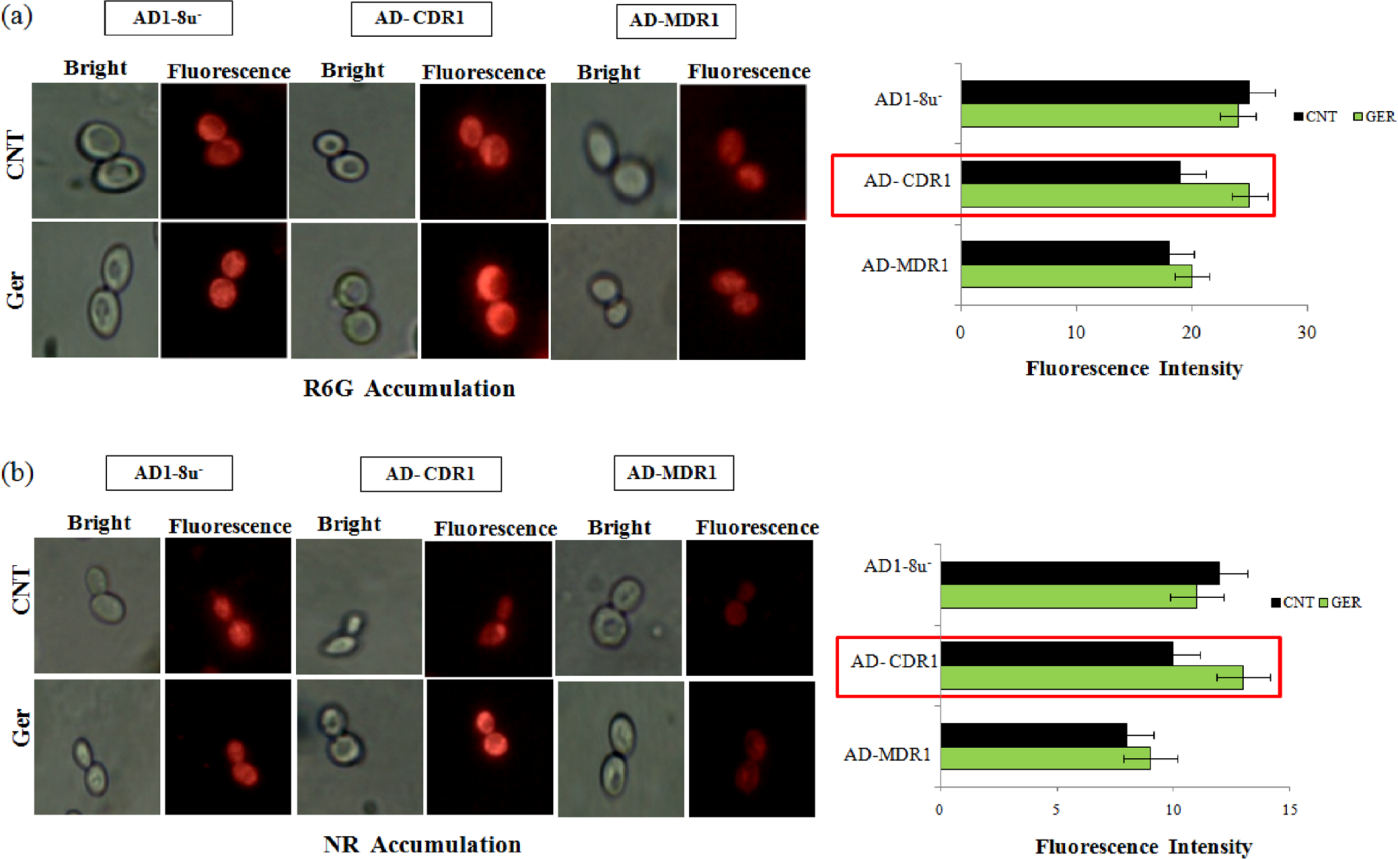
R6G and NR intracellular accumulation. **(a)** Left panel showing the fluorescence images of R6G stained AD1-8u^-^, AD-CDR1, AD-MDR1 strains in presence of Ger (135μg/ml). Right panel shows the bar graph of the fluorescence intensity measured by Image J software. **(b)** Left panel showing the fluorescence images of NR stained AD1-8u^-^, AD-CDR1, AD-MDR1 strains in presence of Ger (135μg/ml). Right panel shows the bar graph of the fluorescence intensity measured by Image J software.

Next, we examined the effect of Ger on efflux pump activities by monitoring the intracellular accumulation of the fluorescent dyes in presence of Ger. Using the AD1-8u^-^ as a control, and Cdr1p (AD-CDR1) and Mdr1p (AD-MDR1) overexpressing strains [39, 40, 41], we estimated the R6G accumulation. Through fluorescence imaging, we found more R6G accumulation due to high fluorescence in case of AD-CDR1 in comparison with the AD-MDR1 strain "Fig 2A". We further confirmed the result with another fluorescent dye NR and accumulation assay showed that intensity was higher in AD-CDR1, projecting that Ger treated cells were unable to efflux the dye "Fig 2B". These observations confirm that Ger specifically inhibits CaCdr1p efflux in *C*. *albicans*.

### Ger competitively inhibits CaCdr1p mediated R6G efflux

**Fig 3:**
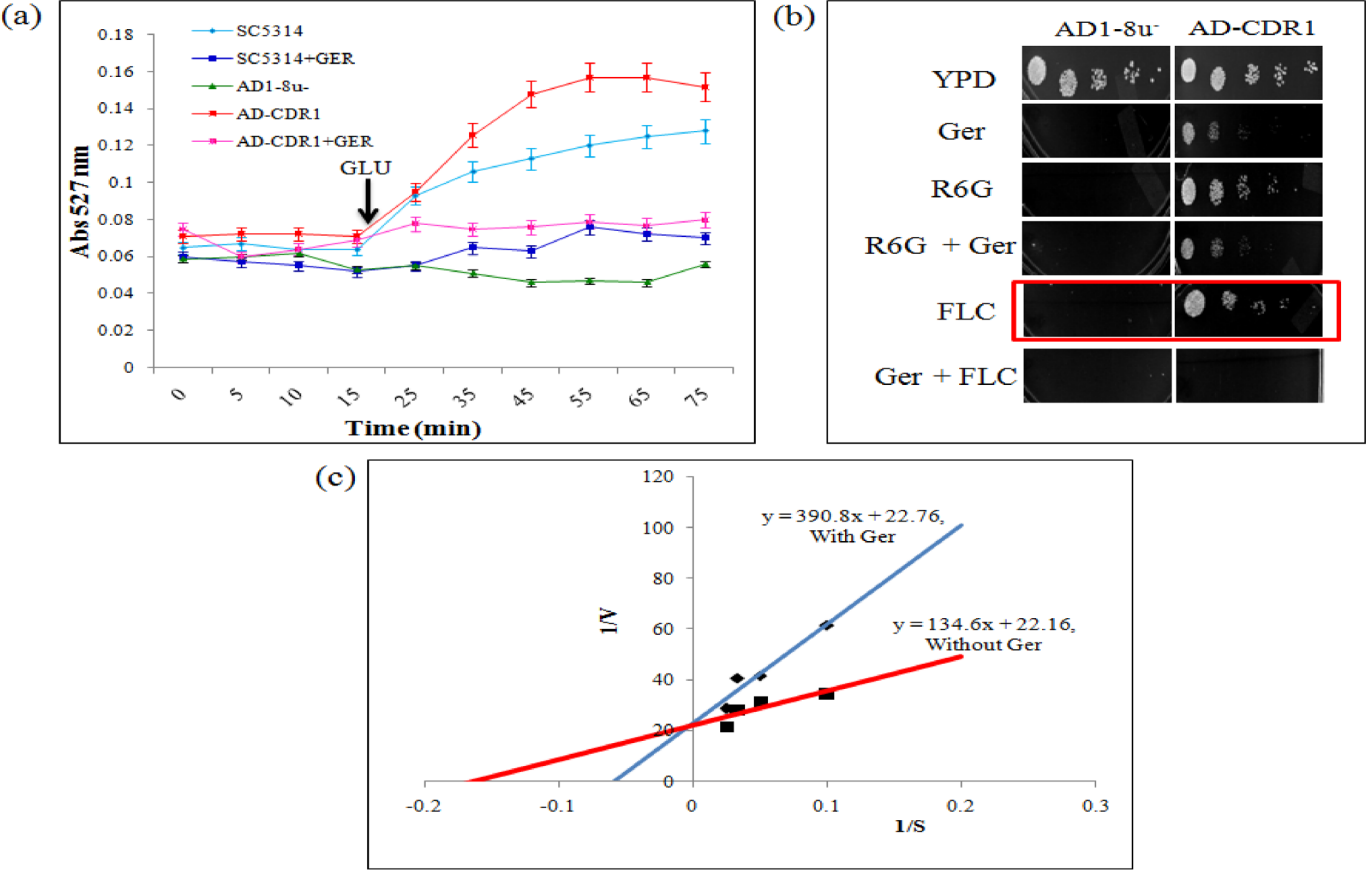
Effect of Ger on R6G efflux. **(a)** Extracellular R6G concentrations in control cells (AD1-8u^-^), cells overexpressing CaCdr1p (AD-CDR1) and Wild-type strain SC5314 of *C*. *albicans*. The energy dependent R6G efflux was initiated by adding glucose (2%; indicated by an arrow) and quantified by measuring the absorbance of the supernatant at 527 nm. The values are the means and standard deviations (indicated by error bars) from three independent experiments. **(b)** Spot assays of AD1-8u^-^ and AD-CDR1 in presence and absence of Ger, R6G and FLC. **(c)** Lineweaver-Burk plot of CaCdr1p-mediated R6G efflux in the presence of Ger. The x axis (1/S) represents the various concentrations (μM) of R6G used, and the y axis (1/V) shows the rate of release of R6G in the presence of Ger (135μg/ml). The rate of each reaction was calculated as nanomoles of R6G released/minute/5X10^6^ cells.

Further, we studied the effect of Ger on CaCdr1p drug efflux activity more closely. Firstly, we performed the R6G efflux to monitor the extracellular R6G efflux in the wild type strain SC5314 [42] in presence of Ger and found that R6G efflux was considerably inhibited in presence of Ger "Fig 3A". To further confirm the specificity on CaCdr1p we again took advantage of CaCdr1p overexpressing strain (AD-CDR1) where AD1-8u^-^ strain was used as negative control and monitored R6G efflux in AD-CDR1. The results showed that there was no efflux of R6G even in energized control AD1-8u^-^ cells as expected while AD-CDR1 showed energy and time dependent efflux of R6G on the addition of glucose which was evident from a steady increase in the extracellular concentration of R6G. Contrary to it, in the Ger treated cells, R6G efflux was inhibited "Fig 3A". Further, we tested through spot assay with AD1-8u^-^ and AD-CDR1 in presence of Ger, FLC and R6G. The result confirmed the specific effect of Ger on CaCdr1 efflux pump "Fig 3B". The inhibited R6G efflux prompted us to further check the type of inhibition by Ger. The Lineweaver-Burk plot clearly revealed that Ger competitively inhibits R6G efflux, with an increase in apparent Km (0.6 to 17.161.5μM) with no effect on the Vmax "Fig 3C".

### Ger binds to active site of CaCdr1p

#### Three dimensional structure prediction and validation

**Fig 4:**
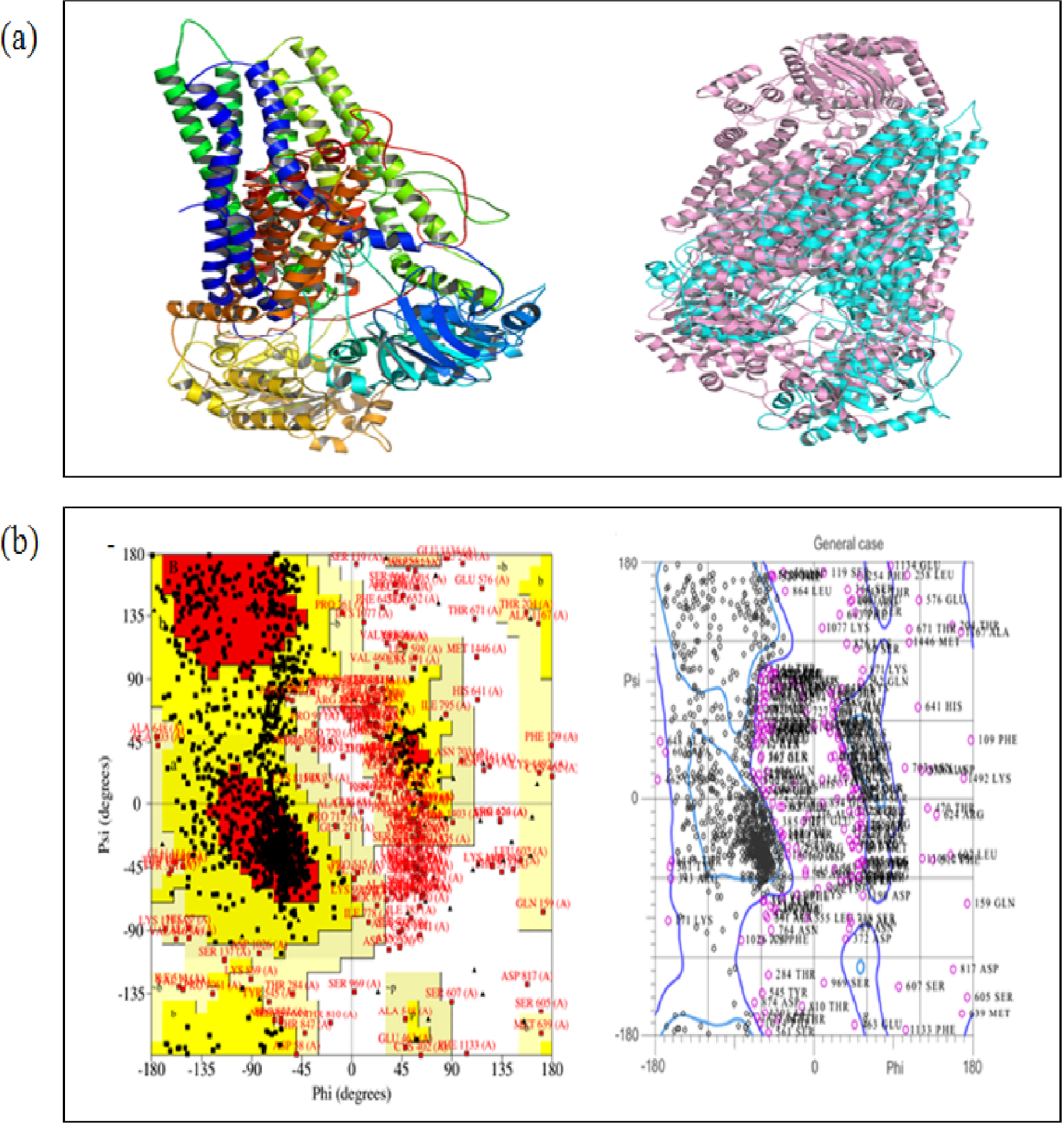
3D structure prediction and validation by I-TASSER. **(a**) Three dimensional structure of *C*. *albicans* CaCdr1p predicted by I-TASSER. Alignment of query protein (pink) with structural analog (cyan) 5do7A in PDB library (right panel). **(b)** Validation of top score model by PROCHECK Ramachandran plot (left panel), MolProbity Ramachandran plot (right panel).

In order to validate the biochemical assays, CaCdr1p model of *C*. *albicans* were predicted through I-TASSER and were obtained in PDB format. I-TASSER server is tool for protein structure and function predictions. It generates high-quality predictions of 3D structure of protein molecules from their amino acid sequences [43].The predicted secondary structure were obtained with confidence score (C-score), solvent accessibility, top ten template from PDB used in alignment, top ten PDB structural analogs, functional analogs protein, and binding site residues. C-score (in a range of -5 to 2) is a parameter to check the quality of predicted models. The higher C-score value denotes the model with better fit [44]. Template modeling score (TM score) also helps to check the quality of protein structure templates by using Global Distance Test (GDT) and MaxSub approaches [45]. The model with C-score -2.07, TM-score 0.47 ± 0.15, and root mean square deviation (RMSD) 15.2 ± 3.4 Å was selected as the best predicted model for CaCdr1p "Fig 4A". Threading templates for query proteins were identified through LOMET meta-server and estimated by normalized Z-score "S3 Table". Generally, if Z-score is >1 it reflects a confident alignment. However, for small sequences it usually fails to show the significant indication of modeling accuracy. Thus, the percentage sequence identity in the threading aligned region (Iden1) and in the whole chain (Iden2) considered for the good homology "S3 Table". The structural alignment program, TM-align, identified 5do7A in PDB library as best structural analog for CaCdr1p with the TM-score of 0.380 "S4 Table".

To check the reliability of the best predicted model, Ramachandran plots were independently obtained from PROCHECK and MolProbity servers. PROCHECK server showed 94.9% (for CaCdr1p) residues were in allowed regions, indicating a good quality model of both proteins "Fig 4B, left panel". MolProbity Ramachandran plot also showed 95.6% residues in allowed regions which again confirmed the quality of predicted model of CaCdr1p "Fig 4B, right panel". Thus both the software justified the reliability of the predicted structure of the proteins.

#### Active site identification

The protuberant binding site of protein CaCdr1 was calculated through CASTp server with ideal parameters "S1 Fig". CASTp evaluation observed the active site amino acids, surface area (388.142), and volume (141.322) of CaCdr1p. In CaCdr1p, all 84 binding pockets were categorized to find the residues about probe 1.4Å radius. The green color denotes the active site amino acid residues involved in formation of binding pockets "S1 Fig".

#### Docking studies

**Fig 5:**
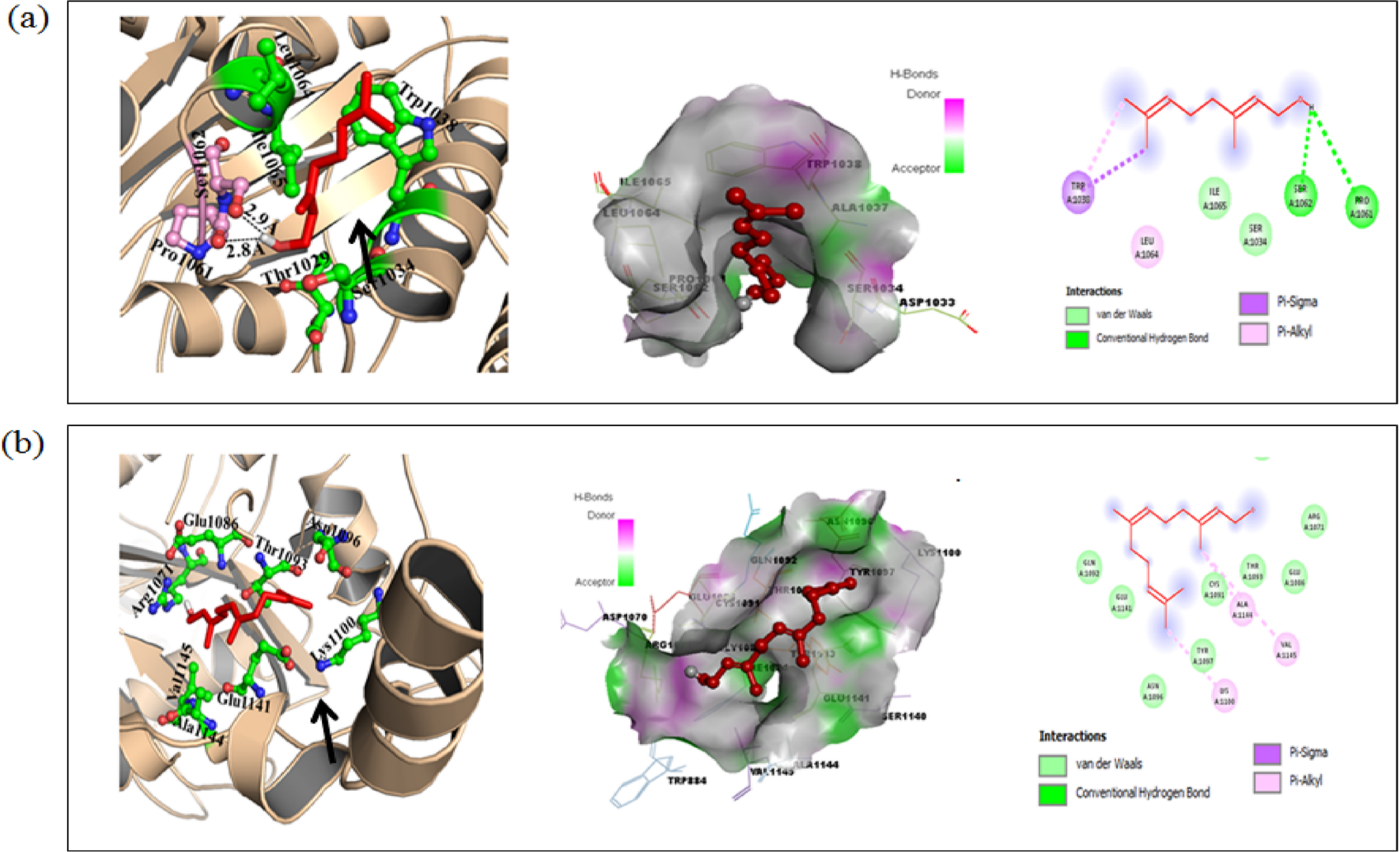
Molecular docking of Ger with CaCdr1p. **(a)** Cartoon model of Cdr1p protein with Ger (left panel), surface view of CaCdr1p with Ger (middle panel) and 2D schematic diagram showing interactions of Ger to the CaCdr1p. Residues involved in hydrogen bonding, Vander Waals interactions, Pi-sigma and Pi-alkyl are represented in different color indicated in inset (right panel). **(b)** Cartoon model of CaCdr1p with Farnesol (known inhibitor) (left panel), surface view of CaCdr1p with Farnesol (middle panel) and 2D schematic diagram showing interactions of Farnesol to the CaCdr1p. Residues involved in hydrogen bonding, Vander Waals interactions, Pi-sigma and Pi-alkyl are represented in different color indicated in inset (right panel).

Auto docking 42 was used to determine the orientation of inhibitors bound in the active site of the CaCdr1p and the conformation with the binding energy value for each molecule was chosen for further analysis and results are given in "Table 1". Our efforts towards the development of new CaCdr1p inhibitors, we investigated the binding modes of CaCdr1p inhibitors using PyMOL software [22]. Results revealed that Ger is nicely bounded into the active site of CaCdr1p with minimum binding energy (∆G) −8.8 kcal/mol as compared to known inhibitor "Table 1". Docking suggests that Ger and Farnesol bind into the active site cavity of CaCdr1p "Fig 5A and 5B, middle panels". Ger formed one H-bond interaction with Pro1061 and Ser1062 of CaCdr1p. Active site residues Leu1025, Thr1029, Ala1037, Cys1056, Ile1054, Cys1056 and Ile1065 of CaCdr1p are involved in hydrophobic interaction with Ger "Fig 5A, left panel". The known inhibitor Farnesol formed no H-bond interaction but hydrophobic interaction with active site residues Arg1071, Glu1086, Thr1093, Asn1096, Lys1100, Glu1141, Ala1144 and Val1145 of CaCdr1p "Fig 5B, left panel".

**Table 1:** Binding energy and specific interaction of CaCdr1p with Ger.

Compounds	Binding energy (kcal/mol)	Protein ligands interaction	
		Hydrophobic	H-bonds
Geraniol	-8.8	Thr1029, Ser1034, Trp1038, Leu1064, Ile1065	Pro1061, Ser1062
Known Inhibitor			
Farnesol	-5.8	Arg1071, Glu1086, Thr1093, Asn1096, Lys1100, Glu1141, Ala1144, Val1145	—

Analysis of docked structure shows that CaCdr1p offers numerous Van der Waals, covalent, Pi-sigma and Pi-alkyl interactions to Ger and Farnesol "Fig 5A and 5B, right panels". Therefore molecular docking, finally concluded that Ger has strong binding affinity with CaCdr1p suggesting a relatively strong binding affinity. The Ger-CaCdr1p docked complex was stabilized by hydrogen bonding as well as Vander Waals, covalent, Pi-sigma and Pi-alkyl interactions.

### Ger does not affect transcription and translation instead causes mislocalization of CaCdr1p

**Fig 6:**
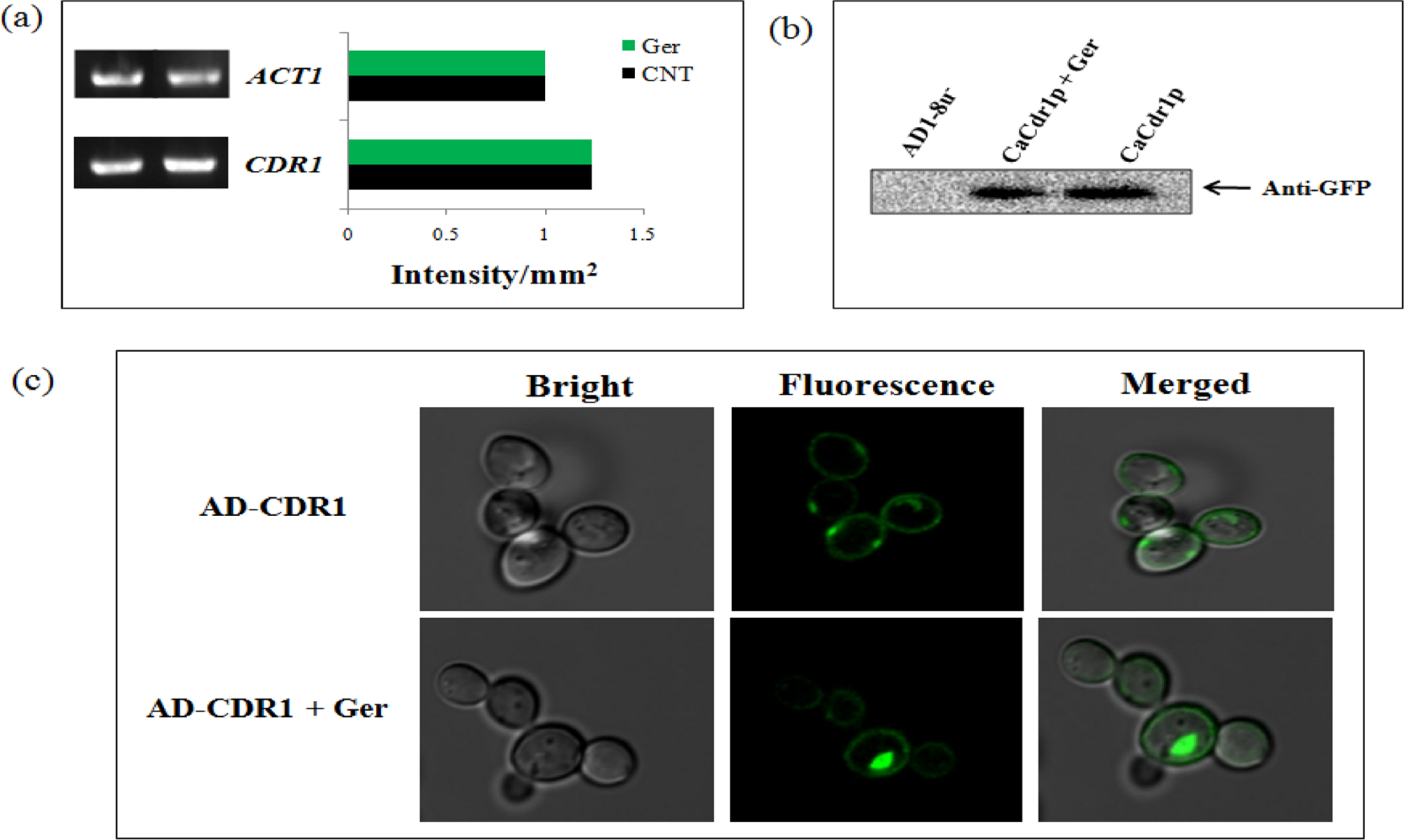
Expression and localization of CaCdr1p in presence of Ger. **(a)** RT-PCR of *CDR1* in response to Ger. The left panel shows transcript levels of the gene in lanes control and Ger treated cells. The right panel shows the quantitation (density expressed as Intensity/mm^2^) of the respective transcript normalized with constitutively expressed *ACT1* transcript. **(b)** Western blot analysis showing the CaCdr1p-GFP protein levels in presence of Ger (sub inhibitory concentration) and developed by anti-GFP antibody. **(c)** Confocal microscopy images showing the membrane mislocalization in the AD-CDR1-GFP tagged strain in presence of Ger.

The virtual screening results and abrogated CaCdr1p activity prompted us to check the change in gene and protein expressions of *CDR1* and CaCdr1p in Ger treated cells. Through RT-PCR, we found no change at transcriptional level in *CDR1* in comparison to constitutively expressed *ACT1* as a control "Fig 6A". Subsequently, we checked the protein expression by performing western blotting and found no change at translational level in CaCdr1p "Fig 6B". Further, we checked the membrane localization of CaCdr1p-GFP in presence of Ger. Interestingly, the confocal microscopy images revealed that CaCdr1p-GFP was properly localized on cell membrane in control cells but was mislocalized in the Ger treated cells "Fig 6C". Additionally we also tested *MDR1* levels and observed that there was no change in *MDR1* and CaMdr1p was also properly localized even in presence of Ger "S3 Fig".

### Ger confers synergism to FLC

**Fig 7:**
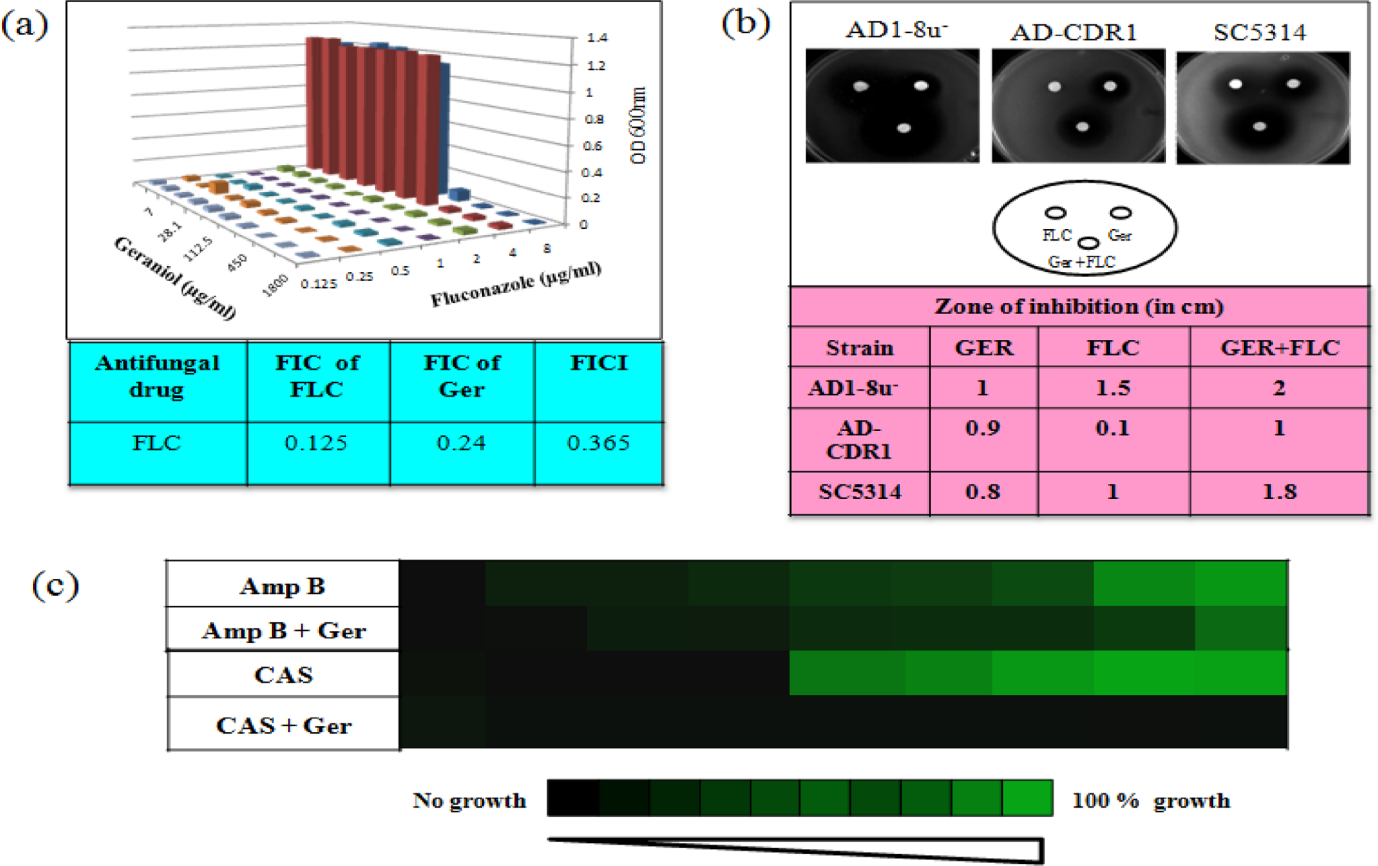
Synergistic effect of Ger with known antifungal drugs. **(a)** Checkerboard assay showing the synergism of Ger with the FLC and FICI was calculated (FICI < 0.5). **(b)** Disk diffusion assay using the AD1-8u^-^, AD-CDR1 and wild type SC5314 by growing them in presence of Ger and FLC along with the combinations. (c) Ger enhances the antifungal efficiency of current therapeutic drugs Amp B and CAS.

We postulated that as an efflux inhibitor, Ger may have synergistic activity with known antifungal drugs. We performed the checkerboard assay of Ger in combination with three classes of known antifungal drugs FLC, Amp B and CAS. Interestingly, the results have shown the synergism with the FLC only as the FICI value of Ger and FLC was found to be less than 0.5 "Fig 7A"while that of Amp B and CAS were greater than 0.5 "S4 Fig". For more validation of the result, we performed the disk diffusion assay to check the combined effect of Ger and FLC on not only SC5314 but AD1-8u^-^ and AD-CDR1 strains. The aureoles around the disk in the YPD plates with drugs clearly reveal the inhibition of *Candida* growth. The zone of inhibition was maximum in the combination of Ger and FLC which confirms the synergism in comparison with their individual effects "Fig 7B". Furthermore, we have also investigated the additive effect of Amp B and CAS with the Ger through checkerboard assay. Interestingly, we found that although Ger was not synergistic with Amp B and CAS yet it does enhanced their efficiencies. We observed that MIC values of Amp B and CAS decreased from 2.5 μg/ml to 62.5 ng/ml and 0.97μg/ml to 62.5 ng/ml respectively in presence of Ger "Fig 7C".

### Ger renders hypersensitivity and ergosterol depletion in FLC resistant clinical isolates

**Fig 8:**
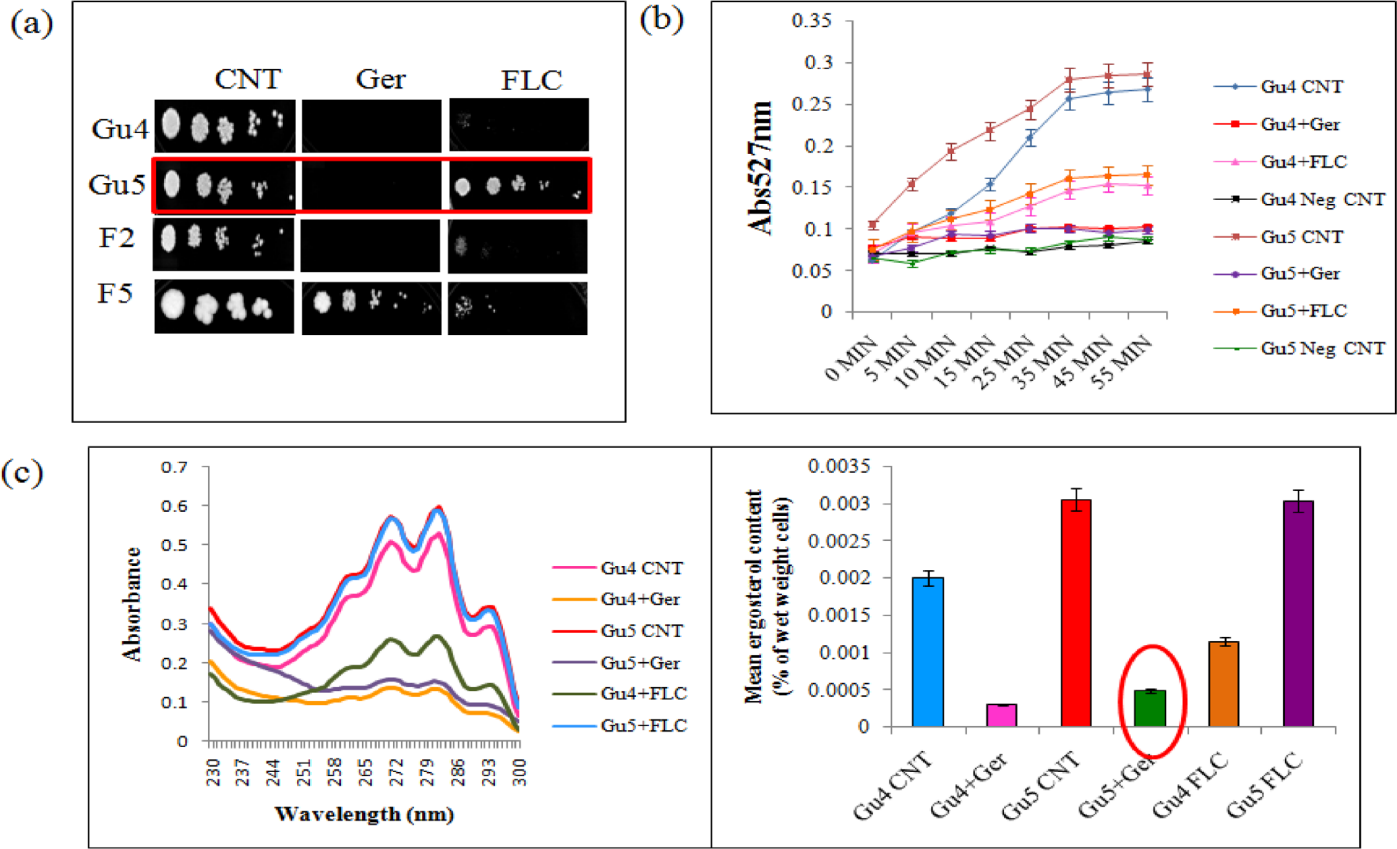
Effect of Ger on FLC resistant clinical isolates. **(a)** Spot assay of Gu4/Gu5 and F2/F5 in presence of Ger (135 μg/mL) and FLC. **(b)** R6G extracellular efflux showing the extracellular R6G concentrations in overexpressing FLC resistant and sensitive match pairs Gu4/ Gu5. The energy dependent R6G efflux was initiated by adding glucose (2%; indicated by an arrow) and quantified by measuring the absorbance of the supernatant at 527 nm. The values are the mean and standard deviations (indicated by error bars) from three independent experiments. **(c)** UV spectrophotometric ergosterol profiles of Gu4/Gu5 strains scanned between 220 and 300 nm in presence of Ger (135 μg/mL) as depicted in left panel. The relative percentage of ergosterol content in the presence of Ger (135μg/mL) is presented in right panel.

The inhibited CaCdr1p efflux activity and FLC synergism led us to make use of drug efflux pumps overexpressing MDR strains. Among them, Gu4 & Gu5 and F2 & F5 are matched pairs of clinical isolates where Gu4 and F2 are sensitive and Gu5 and F5 are the FLC resistant strains due to CaCdr1p and CaMdr1p overexpressions respectively [46, 47, 48]. Firstly, we performed spot assay in presence of Ger and FLC and have found that in presence of Ger, Gu5 showed no growth in comparison with the FLC treated cells. Additionally, we found that F5 strain had no growth defect even in presence of Ger and FLC "Fig 8A". We further monitored R6G efflux on Gu4/Gu5 strains in order to reconfirm the effect of Ger on CaCdr1p efflux. As expected, the results showed the decrease in the extracellular R6G levels in Ger treated Gu4 cells "Fig 8B". Interestingly, in Gu5 cells, we also observed abrogated R6G efflux which was not there in case of FLC treated Gu5 cells reinforcing that Ger specifically affects CaCdr1p "Fig 8B". Furthermore, we quantified ergosterol in the Gu4/Gu5 cells in presence of Ger and FLC. We explored depletion in the ergosterol levels even in Ger treated Gu5 cells whereas no change was observed in FLC treated Gu5 cells "Fig 8C".

### Ger affects mitochondrial membrane potential

**Fig 9:**
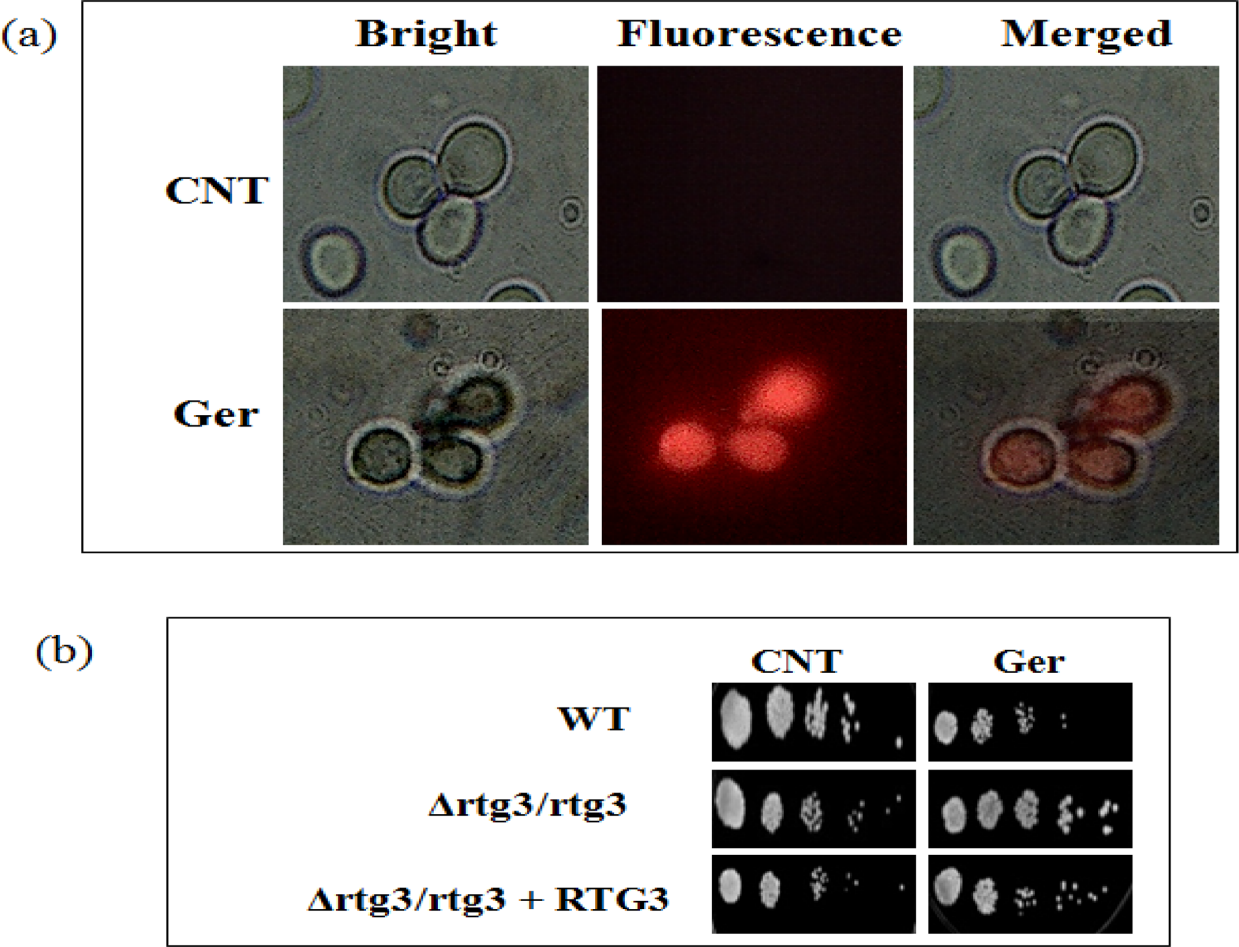
Effect of Ger on mitochondria. **(a)** Fluorescent microscopy images showing Rhodamine B probe for monitoring mitochondrial membrane potential (mtΨm) in the presence of Ger. Scale is 20μm. **(b)** Spot assay demonstrating no growth defect of *C*. *albicans* lacking *RTG3* in presence of Ger (135 μg/mL).

Mitochondrial dysfunction in *C*. *albicans* is known to cause altered susceptibilities to antifungal drugs and defective virulence. In order to monitor the mitochondrial membrane, a florescent dye and mitochondrial probe Rhodamine B was used to determine the change in electrical transmembrane potential by distributing across biological membranes. Interestingly, we found through fluorescence imaging, that Ger treated cells had more red fluorescence cells contrary to no florescence in control cells confirming the effect on mitochondrial potential "Fig 9A". Furthermore, we tested that mitochondrial dysfunction due to Ger involves disruption of retrograde signaling (RTG) signaling. To confirm this, we performed spot assay with *RTG3* mutants [49]. Our results showed no growth inhibition in presence of Ger in the *RTG3* mutants including wild type and revertant strains "Fig 9B".

### Ger inhibits candidal extracellular phospholipase activity, adherence to buccal epithelial cells and biofilm biomass

**Fig 10:**
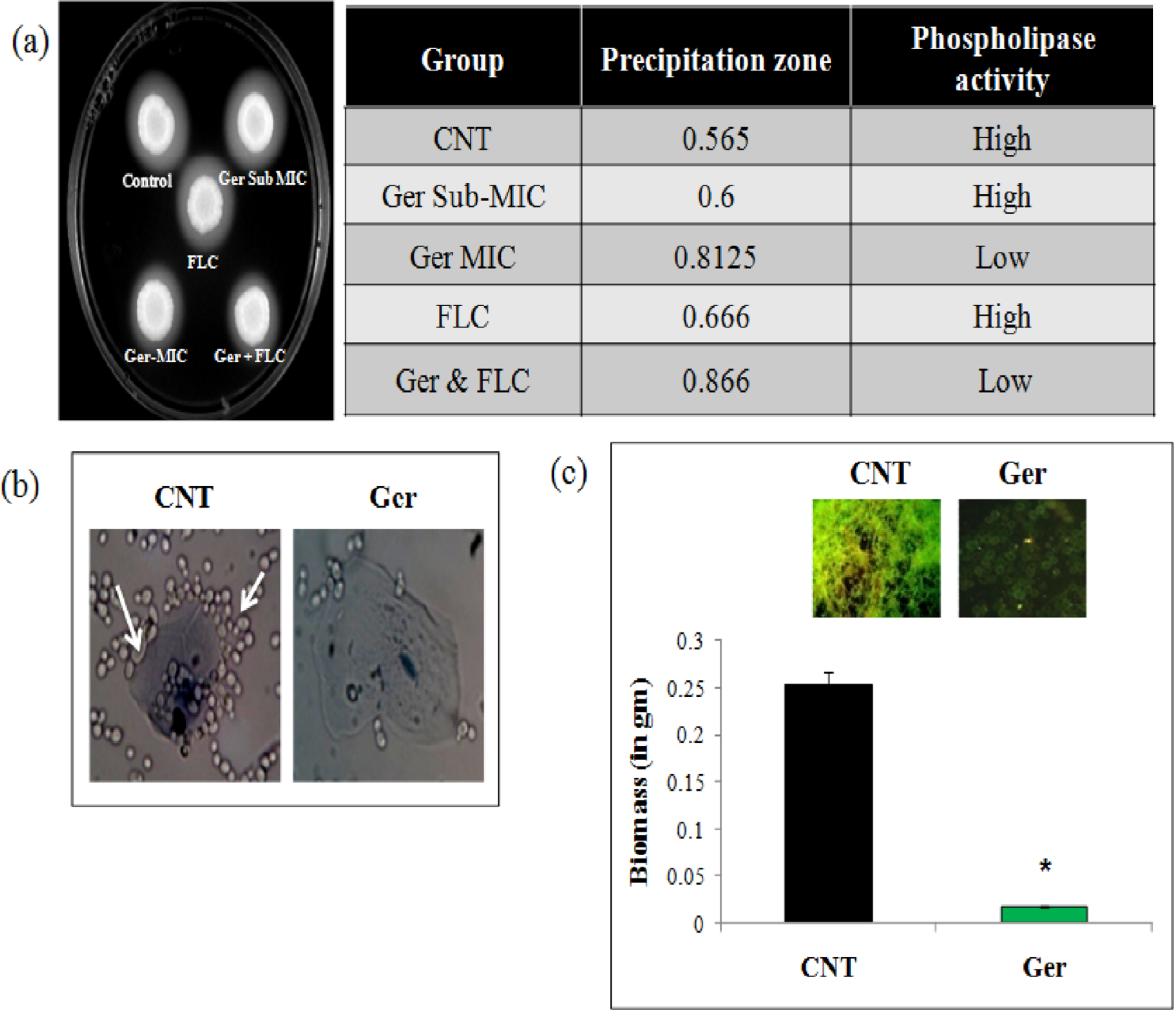
Effect of Ger on virulence markers. **(a)** Extracellular Phospholipase activity and calculation of the precipitation zone represented as the ratio of the diameter of the colony to the cloudy zone plus colony diameter. P <0.05 compared to the control. *C*. *albicans* (90028) with Ger (at SubMIC & MIC values), FLC (1 mg/mL) along with the combinations of Ger and FLC. All data are the averages of triplicate experiments. **(b)** Effect of Ger on cell adherence. The microscopic images have shown the adherence of *Candida* to human buccal epithelial cells in presence of Ger (135 μg/mL). The control cells shows adherence of cells to epithelial cells (depicted by arrow) and Ger treated panel shows non adherence of candidal cells to epithelial cells. **(c)** Effect of Ger on biofilm biomass formed on silicone sheets. Mean of dry weight ± SD of three independent sets of experiments are depicted on Y-axis and * depicts P < 0.05. Inset depicts fluorescence microscopy images of CFW stained biofilms.

The phenotypic virulence marker such as extracellular phospholipase activity was determined by egg yolk method. We observed low phospholipase activity in the Ger treated cells as depicted in "Fig 10A". The calculated Pz (precipitation value) indicates that *C*. *albicans* strain (90028) treated with FLC (1μg/mL) in combination with Ger (135 μg/mL) was 0.866, and at MIC value of Ger (225 μg/mL) alone was 0.8125 indicating a low phospholipase activity. Furthermore, we have checked the effect of Ger on the adherence of candidal cells with the human buccal epithelial cells. The results revealed less adherence in Ger treated candidal cells as compared to untreated cells "Fig 10B". Finally, we evaluated the effect of Ger on the biofilm biomass. Through CFW staining fluorescence microscopy we observed inhibition of biofilm which was further quantified for its biomass. Ger treated cells, had considerable decrease of biomass in comparison with the untreated cells "Fig 10C".

### Ger enhances survival of *C*. *albicans* infected *C*. *elegans*

In order to substantiate the *in-vitro* studies on Ger, we used the nematode model *C*. *elegans* for the evaluation of *in-vivo* antifungal effect of Ger.

#### Ger is non-toxic to *C*. *elegans*

**Fig 11:**
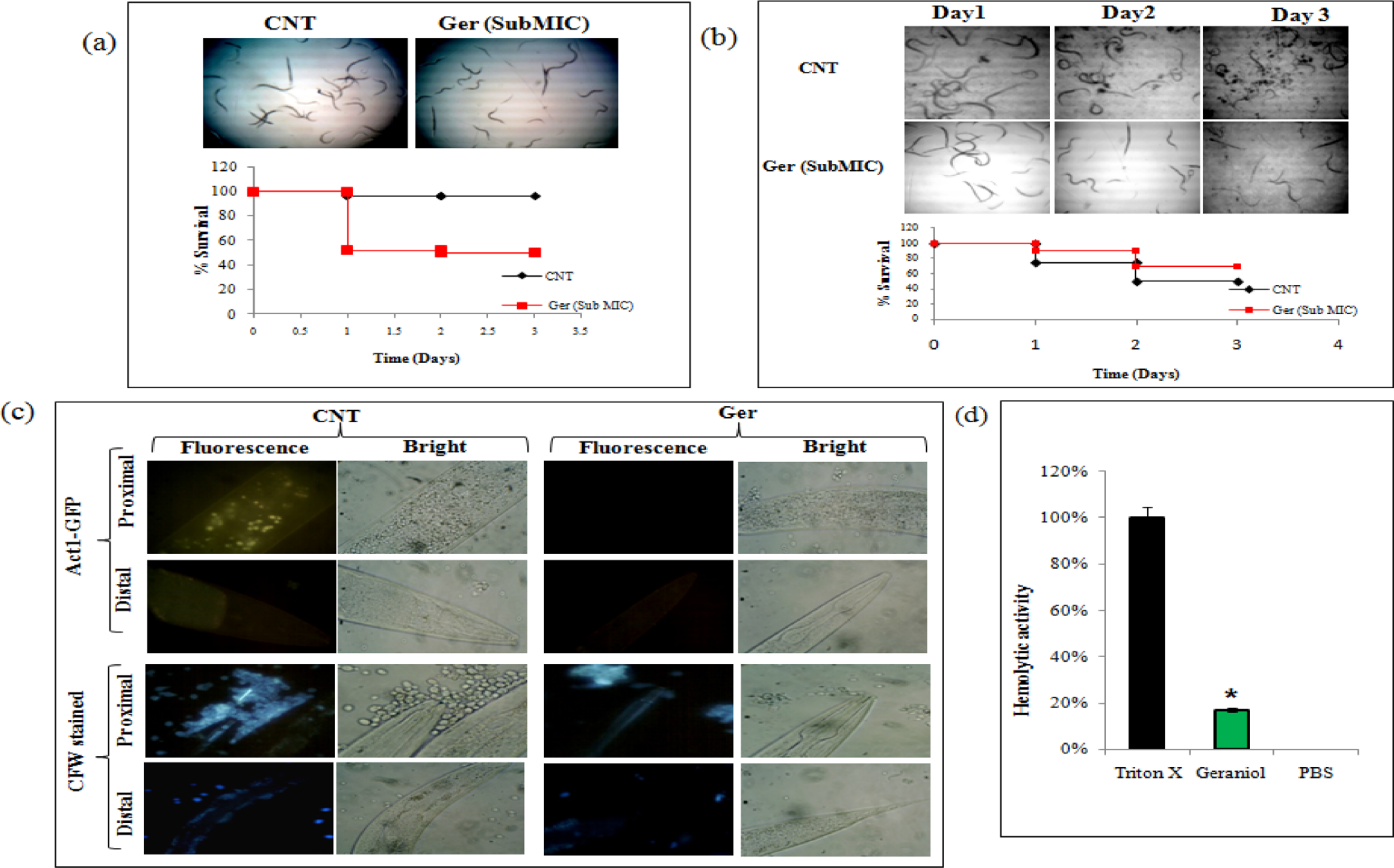
*In-vivo* studies of Ger on nematode model *C*. *elegans*. **(a)** Toxicity test of Ger on *C*. *elegans* depicted by Kaplan–Meier curve showing % survival of *C*. *elegans* in the presence of Ger at sub MIC concentration. Microscopic images (magnification 4x) of nematodes in presence of Ger. Worm survival was determined based on movement (upper panel).The toxicity of Ger was studied on nematodes by determining survival rates after 3 days (lower panel). **(b)** Ger prolongs the survival of *C*. *albicans* infected *C*. *elegans*. Microscopic images showing the survival of infected *C*. *elegans* when treated with Ger on day 1, day2 and day 3. Kaplan–Meier curve showing % survival of *C*. *albicans* infected *C*. *elegans* in the presence of Ger (lower panel). **(c)** Intestinal persistence of *C*. *albicans*. Upper panel shows the Act1p-GFP *C*. *albicans* visualization confirming lower fungal burden in Ger treated worms contrary to untreated nematodes. Lower panel represents the CFW stained *C*. *albicans* visualized in proximal and distal intestine of *C*. *elegans* treated with Ger showing lesser fungal burden. **(d)**. Hemolytic activity of Ger depicted as percentage on Y axis in comparison to the positive control Triton X 100.

First of all, we have checked the toxicity of Ger, for which *C*. *elegans* were grown in presence and absence of Ger. The results have shown that at the sub-MIC concentration (135μg/ml) of Ger, *C*. *elegans* grew at alike pace which looks healthy as untreated control with no appreciable growth defect when observed for three days "Fig 11A". This non toxic concentration of Ger was subsequently used for further evaluation.

#### Ger showed *in-vivo* antifungal activity with *C*. *elegans*

We further investigated the antifungal effect Ger under *in-vivo* condition with *C*. *elegans* infection model. The antifungal activity of Ger was tested at sub-MIC (135μg/ml) for evaluating survival of worms. We observed enhanced survival of *C*. *elegans* in presence of Ger treated *C*. *albicans* in comparison with the untreated cells "Fig 11B".

#### Ger reduces the persistence of *C*. *albicans* in *C*. *elegans*

For examination of fungal encumbrance of *C*. *albicans* in the intestine of *C*. *elegans* we have used the GFP tagged *C*. *albicans* strain Act1p and CFW staining. In the first set of experiment, we used constitutively expressed Act1p-GFP strain of *C*. *albicans* which were fed to *C*. *elegans*. They were monitored for the persistence in the worm intestine. The fluorescent images showed green fluorescence depicting the presence of *C*. *albicans* both in the proximal as well as distal intestine regions in untreated control. Contrary to this, no green fluorescence was observed in worms fed with the Ger (135μg/ml) treated *C*. *albicans cells* "Fig 11C". Furthermore, we used the CFW stained *C*. *albicans* infected *C*. *elegans* which were treated with sub-MIC of Ger and compared with untreated worms. Under fluorescence microscope, blue colored *C*. *albicans* cells were clearly visible in the proximal and distal regions of intestine of untreated *C*. *elegans*. On the other hand, no blue fluorescence was observed in Ger treated worms "Fig 11C". These results clearly indicate that Ger reduces the persistence of *C*. *albicans* in *C*. *elegans*.

#### Ger possess negligible hemolytic activity

Lastly, we also checked the toxicity of Ger by hemolytic assay using the human RBCs. The human RBCs were isolated and subjected to Ger at its MIC concentration. We observed only 20% hemolytic activity in comparison to the Triton-X used as a positive control which causes 100% hemolysis "Fig 11D". This finding further validates low toxicity of Ger.

## Discussion

Candidal infections are one of the major causes of nosocomial infections with high mortality rates worldwide [50]. The known antifungal drugs are either fungistatic or fungicidal in nature. Synthetic drugs FLC and Amp B are mostly fungistatic in nature. In this study, we proposed a monoterpenoid, Ger as fungicidal natural compound "Fig 1". Among the various mechanisms which drive *Candida* to escape from the action of antifungal drugs, the overexpression of drug efflux pumps is the most dominant strategy for drug extrusion. Thus much of the focus remains diverted towards identification of inhibitors/modulators of these drug efflux pumps [51]. In an attempt to identify potential drug transporter inhibitor, we proposed in this study that Ger efficiently targets CaCdr1p. Both ABC and MFS family transporters are the major cause of MDR [9]. Among these drugs efflux transporter families, CaCdr1p and CaMdr1p are the predominant drug efflux transporters which have been studied intrinsically [52]. Our result based on fluorescent dye R6G accumulation revealed Ger to be an inhibitor of CaCdr1p "Fig 2A". To rule out the possibility that Ger also affects CaMdr1p efflux, since R6G is a substrate only for ABC drug transporters, we used NR which is the common substrate of both ABC and MFS families. NR accumulation assay further clarified that Ger specifically affects CaCdr1p and not CaMdr1p "Fig 2B". Herein we have provided the first evidence that Ger can reverse the extrusion of drugs mediated exclusively by ABC drug transporter CaCdr1p since the efflux mediated by CaMdr1p could not be inhibited by Ger. This specificity of Ger towards particular class of ABC superfamily is not uncommon. For instance, curcumin, quorum sensing molecule farnesol, beauvericin and styrylquinolines affect efflux activities only of ABC drug transporters [12, 24, 28, 53]. Similarly, palmarumycin P3 and phialocephalarin B and jatrophane derivatives selectively affect CaMdr1p activities [11, 54]

For further insights, we performed the R6G drug efflux since R6G is a specific substrate of CaCdr1p transporter. The results revealed that Ger significantly inhibits the R6G efflux "Fig 3A". To further validate the abrogated efflux, we took advantage of overexpressing CaCdr1p strains. Expectedly, we observed that there was no efflux in AD1-8u^-^ strain and maximum efflux in AD-CDR1. Contrary, in presence of Ger even efflux in AD-CDR1 was inhibited "Fig 3A"reinforcing that Ger specifically inhibits CaCdr1p activity "Fig 3A". This was also consistent with spot assays which revealed that AD-CDR1 grew well in presence of R6G and FLC but in presence of Ger the cells were sensitive "Fig 3B". To further investigate the type of inhibition, we performed the enzyme kinetic studies using the Ger and R6G as substrates. Interestingly, Ger competitively inhibits the CaCdr1p transporter efflux activity as there was no change in Vmax but apparent Km increases "Fig 3C".

For further validation of our results, we have used the advanced bioinformatics tools, homology modeling and docking studies which provided more evidence on the effect of Ger on CaCdr1p. The results showed that Ger binds into the active site cavity of CaCdr1p "Fig 4" which was indicated by excellent minimum binding energy in comparison to the known inhibitor farnesol "Table 1". Curcumin is another natural compound apart from quorum sensing molecule farnesol which also competitively inhibits the CaCdr1p transporter efflux activity [12, 24]. Our results also correlates with another study where β-estradiol also competitively inhibits R6G efflux by binding to similar active cites of CaCdr1p where R6G binds [55]. However, elaborate binding studies need to be performed to resolve whether or not Ger is substrate for CaCdr1p. Docking studies also showed that Ger binding to the CaCdr1p transporter is also due to the hydrogen bonding where as farnesol doesn't indicated any hydrogen bonding. The different kinds of interactions including Van der Waals, covalent bonding, hydrophobic binding of Ger with the amino acids of active site of CaCdr1p "Fig 5". These results strongly support that Ger has peculiar binding affinity with the CaCdr1p active site which validates our above biochemical assay showing that Ger competitively inhibits CaCdr1p activity. We further hypothesized that Ger might have any effect on transcriptional or translational levels of *CDR1* and CaCdr1p respectively. Our results however depicted no change at either transcript "Fig 6A" or protein expression levels "Fig 6B" respectively. On the contrary, we explored the membrane mislocalization of CaCdr1p in GFP tagged strain in presence of Ger "Fig 6C". This could be due to the fact that Ger binding to CaCdr1p may induce certain conformational changes leading to the missorting.

Many natural compounds have been tested for the synergism or additive effects with the known antifungal drugs [11]. In this study, we also tested the synergism with the known drugs FLC, Amp B and CAS. Interestingly, Ger was found to be synergistic with FLC "Fig 7A" but not with other drugs "S4 Fig". FLC synergism was also validated by disk diffusion assay which correlates well by using AD-CDR1 "Fig 7B". Coincidently, we also found that even though Ger does not show synergy with Amp B and CAS, it increases the efficacy of Amp B and CAS "Fig 7C". We further extended the study to examine the outcome of abrogated CaCdr1p efflux and FLC synergism in presence of Ger by using matched pairs of FLC resistant clinical isolates. We used two such pairs namely Gu4 & Gu5 and F2 & F5 which are FLC sensitive and resistant pairs due to overexpressions of CaCdr1p and CaMdr1p respectively [46, 47, 48]. We found that Ger could sensitize FLC resistant Gu5 but not F5 which corroborates the fact that Ger specifically inhibits CaCdr1p but not CaMdr1p "Fig 8A". Gu4 & Gu5 strains were also evaluated for R6G efflux and expectedly we observed that even in Gu5 strain R6G efflux was inhibited in presence of Ger contrary to FLC "Fig 8B". We further estimated the ergosterol levels in Gu4 & Gu5 since ergosterol is the primary target of FLC. Interestingly, we observed that ergosterol levels were declined even in Gu5 FLC resistant strain which could be the causal reason for reversal of drug resistance "Fig 8C". Based on these findings, our hypothesis that Ger specifically inhibits CaCdr1p and not CaMdr1p activity may be attributed to the fact that both these transporters display different lipid specificities and CaCdr1p is targeted to membrane rafts region rich in ergosterol [56] which gets depleted in presence of Ger.

Mitochondria dysfunctioning is known to influence drug susceptibilities and virulence traits of *C*. *albicans* [57, 58]. Moreover CaCdr1p activity is known to be also influenced by mitochondria [59]. These facts prompted us to investigate the mitochondria membrane potential with the help of fluorescent dye Rhodamine B which clearly depicted the effect on the mitochondrial membrane potential due to enhance fluorescence "Fig 9A". Interestingly, RTG signaling which again is known to be associated with drug resistance seems independent from the effect of Ger because the growth did not get hampered for the *RTG3* mutant even in the presence of Ger "Fig 9B".

In this study, we have also extended our work to check fungal virulence markers which are attractive potential targets for drug development. Phospholipase enzymes have been secreted by *Candida* for invasion and degradation of the host tissues by colonization and infection [60]. Extracellular phospholipase lyses host cells to facilitate adhesion and penetration by breaking down the phospholipids of the cell membrane causing cell lysis, thereby causing direct host cell damage and lysis which is proposed as a major mechanism contributing to *Candida* virulence. We found that the phospholipase activity of Ger at MIC was less in comparison to control. Similarly, the combined treatment of Ger and FLC decreased the phospholipase activity in *Candida* in comparison to FLC alone "Fig 10A". Since increased cell adherence is known to increase the phospholipase activity hence we further assessed the cell adherence on human buccal epithelial cells which was considerably reduced in presence of Ger "Fig 10B". Since adherence is the first step towards biofilm formation hence we also estimated the biofilm biomass which was diminished in presence of Ger "Fig 10C". Thus Ger could be a potential inhibitor of *Candida* virulence factors.

Lastly, the *in-vivo* studies with nematode model *C*. *elegans* further verified the effectiveness and non toxic nature of Ger "Fig 11A". It also confirmed that worms survival rate were increased in presence of Ger by inhibiting the growth of candidal cells "Fig 11B". The results also indicated that Ger increased the intestinal persistence of *C*. *elegans* when fed with the Ger treated *C*. *albicans* "Fig 11C". The low hemolytic activity has also confirmed the non toxic nature of Ger on human RBCs "Fig 11D".

**Fig 12:**
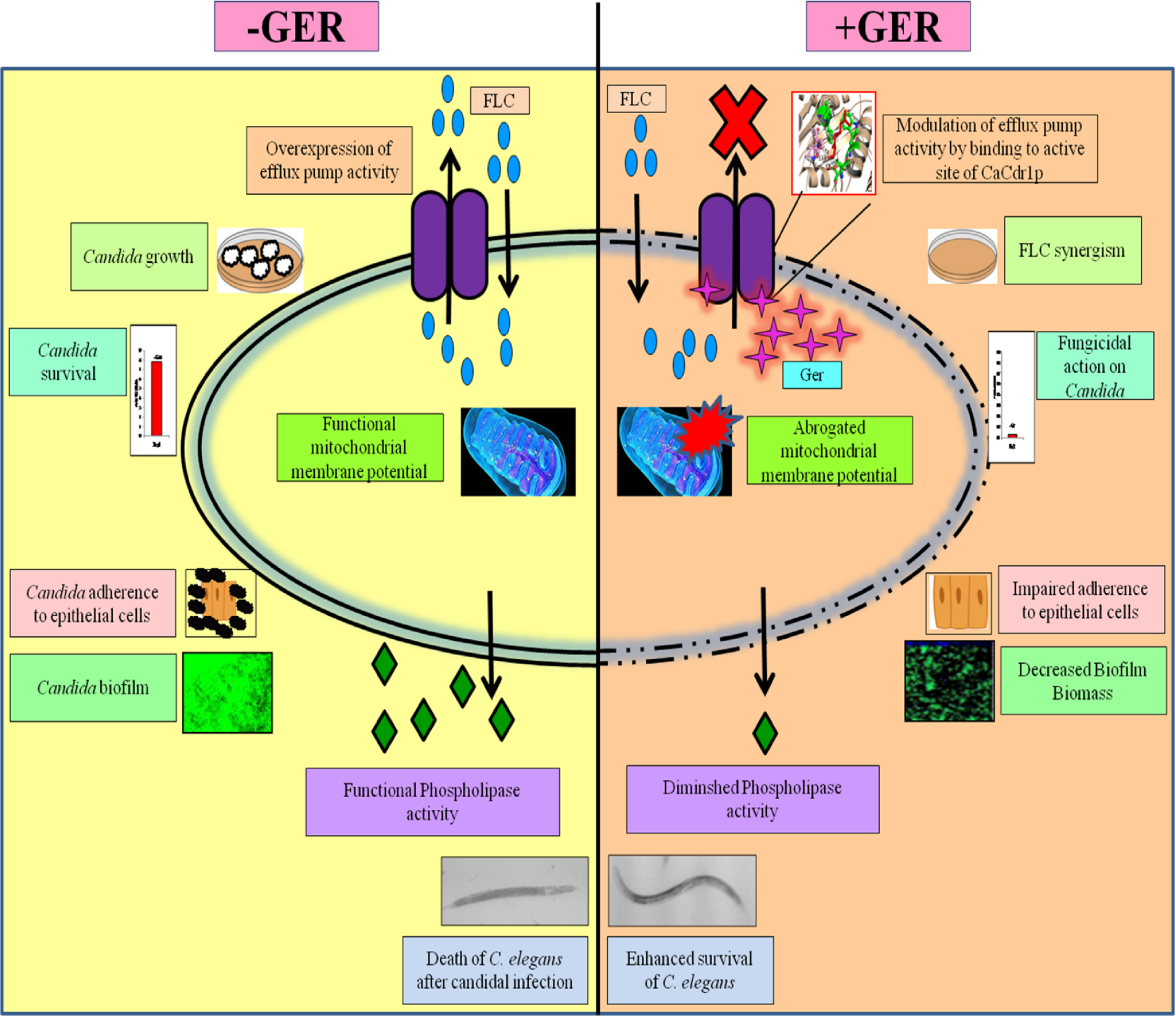
Schematic proposal of the multi-target activity of Ger.

Taken together, we have demonstrated that Ger is fungicidal in nature that modulates the CaCdr1p efflux pump activity by binding to its active site which was further supported by docking studies. The ability of Ger to sensitize *Candida* cells to FLC opens up new options for combination therapy "Fig 12". The non toxic nature of Ger along with the promising *in-vivo* results further strengthens its candidature for continued future investigations.

## Supporting information

S1 Fig Binding pocket identification by CASTp server. Upper panel shows green color boxes highlights the amino acid residues present in the binding site. Lower panel shows the binding sites of CaCdr1p.
(DOC)

S2 Fig UV spectra of RNA isolated from control and Ger treated *C*. *albicans* cells. UV spectra of RNA isolated from control and Ger treated *C*. *albicans* cells.
(DOC)

S3 Fig Expression and localization of CaCdr1p in presence of Ger. (a) RT-PCR of *MDR1* in response to Ger. **(a)** The left panel shows transcript level of the gene in lanes control and Ger treated cells. The right panel shows the quantitation (density expressed as Intensity/mm^2^) of the respective transcript normalized with constitutively expressed *ACT1* transcript. **(b)** Confocal microscopy images showing proper membrane localization in the AD-MDR1-GFP tagged strain in presence of Ger.
(DOC)

S4 Fig Non Synergistic effect of Ger with known antifungal drugs. Checkerboard assay showing the no synergism of Ger with the CAS & Amp B and FICI was calculated (FICI > 0.5).
(DOC)

S1 Table List of strains used in the study. (DOC)

S2 Table List of primers used in the study. (DOC)

S3 Table Top ten templates used by I-TASSER for threading alignment. (DOC)

S4 Table Top ten structural analogs in PDB identified by TM-align. (DOC)
