# Supplementary material for: Fungicidal action of geraniol against Candida albicans is potentiated by abrogated CaCdr1p drug efflux and fluconazole synergism
Source: PLoS One. 2018 Aug 29;13(8):e0203079. doi: 10.1371/journal.pone.0203079 (PMC6114893; doi:10.1371/journal.pone.0203079)
Supplement: S1 Fig — Upper panel shows green color boxes highlights the amino acid residues present in the binding site. Lower panel shows the binding sites of CaCdr1p. (DOC) [file pone.0203079.s001.doc]

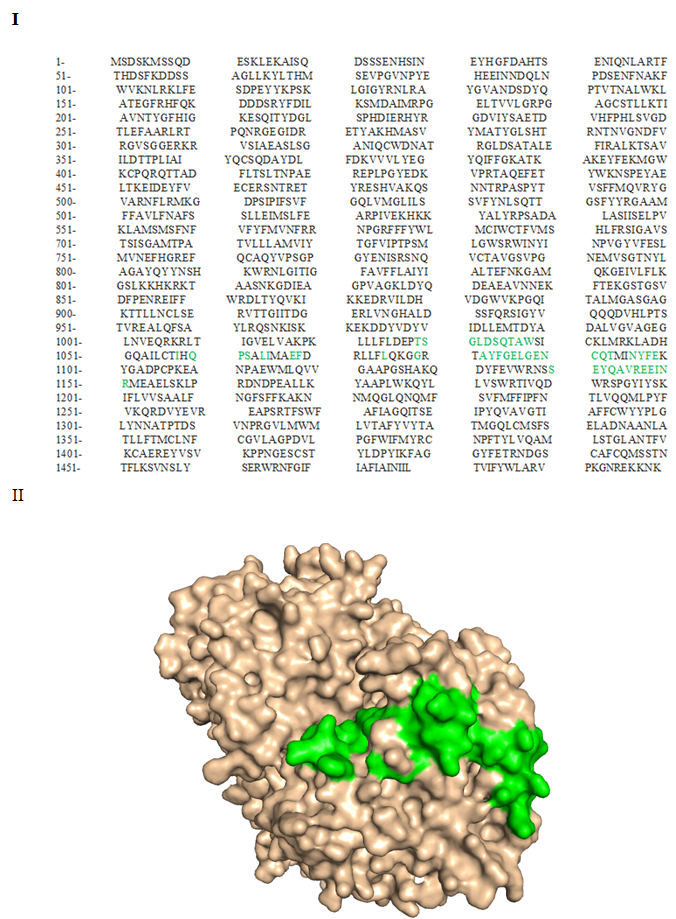


**S1 Fig**: **Binding pocket identification by CASTp server**. Upper panel shows green color boxes highlights the amino acid residues present in the binding site. Lower panel shows the binding sites of CaCdr1p.
