# Supplementary material for: Fungicidal action of geraniol against Candida albicans is potentiated by abrogated CaCdr1p drug efflux and fluconazole synergism
Source: PLoS One. 2018 Aug 29;13(8):e0203079. doi: 10.1371/journal.pone.0203079 (PMC6114893; doi:10.1371/journal.pone.0203079)
Supplement: S2 Fig — UV spectra of RNA isolated from control and Ger treated C. albicans cells. (DOC) [file pone.0203079.s002.doc]

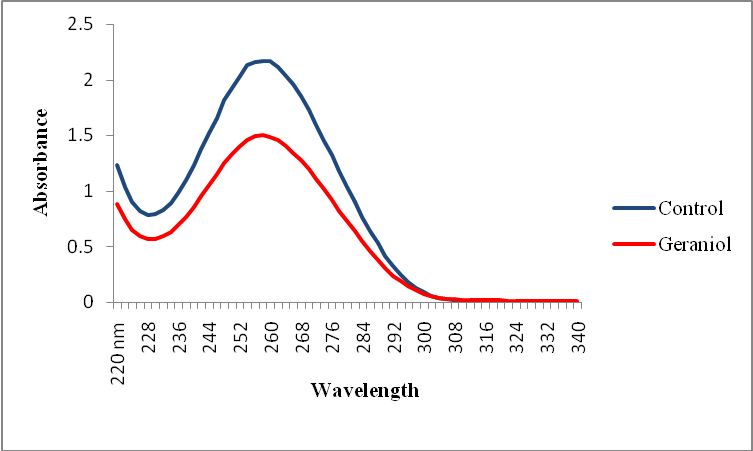


A_260_/A_280_ of Control samples: 2.098

A_260_/A_280_ of Ger treated samples: 2.054

**S2 Fig: UV spectra of RNA isolated from control and Ger treated *C. albicans* cells.**
