# Supplementary material for: Fungicidal action of geraniol against Candida albicans is potentiated by abrogated CaCdr1p drug efflux and fluconazole synergism
Source: PLoS One. 2018 Aug 29;13(8):e0203079. doi: 10.1371/journal.pone.0203079 (PMC6114893; doi:10.1371/journal.pone.0203079)
Supplement: S3 Fig — (a) RT-PCR of MDR1 in response to Ger. (a) The left panel shows transcript level of the gene in lanes control and Ger treated cells. The right panel shows the quantitation (density expressed as Intensity/mm2) of the respective transcript normalized with constitutively expressed ACT1 transcript. (b) Confocal microscopy images showing proper membrane localization in the AD-MDR1-GFP tagged strain in presence of Ger. (DOC) [file pone.0203079.s003.doc]

a)

b)

**S3 Fig:** **Expression and localization of CaMdr1p in presence of Ger**. (a) RT-PCR of *MDR1* in response to Ger. **(a)** The left panel shows transcript level of the gene in lanes control and Ger treated cells. The right panel shows the quantitation (density expressed as Intensity/mm^2^) of the respective transcript normalized with constitutively expressed *ACT1* transcript. **(b)** Confocal microscopy images showing proper membrane localization in the AD-MDR1-GFP tagged strain in presence of Ger.
