# Supplementary material for: Fungicidal action of geraniol against Candida albicans is potentiated by abrogated CaCdr1p drug efflux and fluconazole synergism
Source: PLoS One. 2018 Aug 29;13(8):e0203079. doi: 10.1371/journal.pone.0203079 (PMC6114893; doi:10.1371/journal.pone.0203079)
Supplement: S4 Fig — Checkerboard assay showing the no synergism of Ger with the CAS & Amp B and FICI was calculated (FICI > 0.5). (DOC) [file pone.0203079.s004.doc]

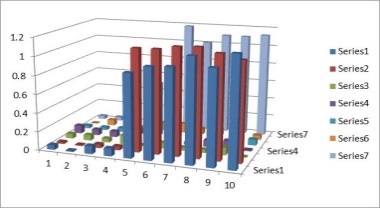
 **
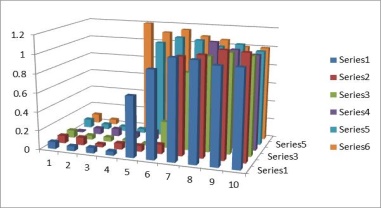
**

**Amp B CAS**

| **Antifungal drug** | **FIC of antifungal drug** | **FIC of Ger** | **FICI** |
| --- | --- | --- | --- |
| **Amp B** | **0.5** | **0.22** | **0.72** |
| **CAS** | **0.5** | **0.20** | **0.70** |

**S4 Fig: Non Synergistic effect of Ger with known antifungal drugs.** Checkerboard assay showing the no synergism of Ger with the CAS & Amp B and FICI was calculated (FICI > 0.5).
