# Supplementary material for: Fungicidal action of geraniol against Candida albicans is potentiated by abrogated CaCdr1p drug efflux and fluconazole synergism
Source: PLoS One. 2018 Aug 29;13(8):e0203079. doi: 10.1371/journal.pone.0203079 (PMC6114893; doi:10.1371/journal.pone.0203079)
Supplement: S1 Table — (DOC) [file pone.0203079.s005.doc]

**S1** Table: List of strains used in this study

| **Name** | **Strain** | **Genome** | **Reference** |
| --- | --- | --- | --- |
| *C. albicans* | SC5314 | Reference strain | 42 |
| *C. albicans* | 90028 | Reference strain | 34 |
| *C. albicans* (WT) | HBCA16 | BWP17 | 49 |
| *C. albicans*  *(Δrtg3/Δrtg3)* | HBCA228 | BWP17 rtg3::HIS1/rtg3::ARG4+CIP10 | 49 |
| *C. albicans*  *(Δrtg3/RTG3)* | HBCA231 | BWP17 rtg3::HIS1/rtg3::ARG4+CIP10-CaRTG3 | 49 |
| AD1-8u^-^_ |  | MAT_ *pdr1*-*3 his1 ura* _*yor1*::*hisG* _*snq2*::*hisG* _*pdr5*::*hisG* _*pdr10*::*hisG*  _*pdr11*::*hisG* _*ycf1*::*hisG pdr3*::*hisG* _*pdr15*::*hisG* | 39 |
| AD-CDR1 |  | AD1-8u^-^ cells harboring the CaCDR1-GFP ORF integrated at the PDR5 locus | 40 |
| AD-CaMDR1 |  | AD1-8u^-^ cells harboring the CaMDR1-GFP ORF integrated at the PDR5 locus | 41 |
| Gu4 |  | FLC-sensitive clinical isolate | 46 |
| Gu5 |  | FLC-resistant clinical isolate overexpressing *CDR1* & *CDR2* | 46 |
| F2 |  | FLC-sensitive clinical isolate | 47 |
| F5 |  | FLC-resistant clinical isolate overexpressing *MDR1* | 47 |
