# Supplementary material for: Fungicidal action of geraniol against Candida albicans is potentiated by abrogated CaCdr1p drug efflux and fluconazole synergism
Source: PLoS One. 2018 Aug 29;13(8):e0203079. doi: 10.1371/journal.pone.0203079 (PMC6114893; doi:10.1371/journal.pone.0203079)
Supplement: S2 Table — (DOC) [file pone.0203079.s006.doc]

**S2 Table: List of primers used for RT–PCR in the study.**

| **Sr. No.** | **Gene name** | **Primer Sequence** |
| --- | --- | --- |
|  | *ACT1* | **F:**TTTTGACCTTGAGATACCCA  **R:**GGAGCTCTGAATCTTTCGTT |
|  | *CDR1* | **F:**GGAGTTTGGGTGCTGTTTGT  **R**:AATTCAACCCCAATGGTCAA |
|  | *MDR1* | **F**:GGAGTTTGGGTGCTGTTTGT  **R**:TGTGGTACCCAATTCAACGA |
