# Supplementary material for: Fungicidal action of geraniol against Candida albicans is potentiated by abrogated CaCdr1p drug efflux and fluconazole synergism
Source: PLoS One. 2018 Aug 29;13(8):e0203079. doi: 10.1371/journal.pone.0203079 (PMC6114893; doi:10.1371/journal.pone.0203079)
Supplement: S3 Table — (DOC) [file pone.0203079.s007.doc]

**S3 Table: Top ten templates used by I-TASSER for threading alignment.**

| **Rank** | **PDB Hit** | **Iden1** | **Iden2** | **Cov.** | **Z-score** |
| --- | --- | --- | --- | --- | --- |
| **1** | **5do7A** | **0.22** | **0.11** | **0.38** | **2.58** |
| **2** | **5do7A** | **0.22** | **0.11** | **0.38** | **5.36** |
| **3** | **5nj3A** | **0.26** | **0.11** | **0.37** | **3.01** |
| **4** | **5do7A** | **0.21** | **0.11** | **0.39** | **2.37** |
| **5** | **5do7A** | **0.23** | **0.11** | **0.38** | **2.19** |
| **6** | **3w3tA** | **0.09** | **0.12** | **0.66** | **2.28** |
| **7** | **5do7A** | **0.24** | **0.11** | **0.38** | **2.43** |
| **8** | **5do7A** | **0.22** | **0.11** | **0.38** | **2.57** |
| **9** | **5do7A** | **0.22** | **0.11** | **0.38** | **4.21** |
| **10** | **5do7B** | **0.22** | **0.10** | **0.37** | **3.73** |

Ident1 is the percentage sequence identity of the templates in the threading aligned region with the query sequence.

Ident2 is the percentage sequence identity of the whole template chains with query sequence.

Cov represents the coverage of the threading alignment and is equal to the number of aligned residues divided by the length of query protein.

Z-score is the normalized Z-score of the threading alignments. Alignment with a Normalized Z-score >1 mean a good alignment and vice versa.
