# Supplementary material for: Fungicidal action of geraniol against Candida albicans is potentiated by abrogated CaCdr1p drug efflux and fluconazole synergism
Source: PLoS One. 2018 Aug 29;13(8):e0203079. doi: 10.1371/journal.pone.0203079 (PMC6114893; doi:10.1371/journal.pone.0203079)
Supplement: S4 Table — (DOC) [file pone.0203079.s008.doc]

**S4 Table: Top ten structural analogs in PDB identified by TM-align.**

| **Rank** | **PDB Hit** | **TM-score** | **RMSD^a^** | **IDEN^a^** | **Cov.** |
| --- | --- | --- | --- | --- | --- |
| **1** | **5do7A** | **0.380** | **0.76** | **0.219** | **0.381** |
| **2** | **5nj3A** | **0.355** | **2.80** | **0.241** | **0.371** |
| **3** | **2pffH** | **0.259** | **10.36** | **0.037** | **0.402** |
| **4** | **2uvcH** | **0.259** | **9.71** | **0.034** | **0.382** |
| **5** | **3hmjG** | **0.259** | **10.40** | **0.032** | **0.403** |
| **6** | **2vkzG** | **0.258** | **10.18** | **0.023** | **0.394** |
| **7** | **6bq1A** | **0.238** | **10.38** | **0.041** | **0.372** |
| **8** | **2vz9B** | **0.233** | **10.44** | **0.024** | **0.365** |
| **9** | **5x6oC** | **0.231** | **9.80** | **0.036** | **0.345** |
| **10** | **4bedB** | **0.230** | **10.05** | **0.028** | **0.352** |

TM-score is a recently proposed scale for measuring the structural similarity between two structures

RMSD^a^ is the RMSD between residues that are structurally aligned by TM-align.

IDEN^a^ is the percentage sequence identity in the structurally aligned region.

Cov represents the coverage of the alignment by TM-align and is equal to the number of structurally aligned residues divided by length of the query protein.
